# Different Response of *Ptch* Mutant and *Ptch* Wildtype Rhabdomyosarcoma Toward SMO and PI3K Inhibitors

**DOI:** 10.3389/fonc.2018.00396

**Published:** 2018-09-25

**Authors:** Natalie Geyer, Rosalie Ridzewski, Julia Bauer, Maria Kuzyakova, Kai Dittmann, Christian Dullin, Albert Rosenberger, Hans-Ulrich Schildhaus, Anja Uhmann, Simone Fulda, Heidi Hahn

**Affiliations:** ^1^Institute for Human Genetics, University Medical Center Goettingen, Goettingen, Germany; ^2^Institute for Celluar and Molecular Immunology, University Medical Center Goettingen, Goettingen, Germany; ^3^Institute for Diagnostic and Interventional Radiology, University Medical Center Goettingen, Goettingen, Germany; ^4^Department of Genetic Epidemiology, University Medical Center Goettingen, Goettingen, Germany; ^5^Institute for Pathology, University Medical Center Goettingen, Goettingen, Germany; ^6^Institute for Experimental Cancer Research in Pediatrics, Goethe-University Frankfurt, Frankfurt, Germany

**Keywords:** ERMS, HH, PTCH, PI3K, vismodegib, sonidegib, HhAntag, pictilisib

## Abstract

Rhabdomyosarcoma (RMS) is the most common pediatric soft tissue sarcoma with poor prognosis. RMS frequently show Hedgehog (HH) pathway activity, which is predominantly seen in the embryonal subtype (ERMS). They also show activation of Phosphatidylinositol-4,5-bisphosphate 3-kinase (PI3K) signaling. Here we compared the therapeutic effectiveness and the impact on HH target gene expression of Smoothened (SMO) antagonists with those of the PI3K inhibitor pictilisib in ERMS with and without mutations in the HH receptor *Patched1* (*PTCH*). Our data demonstrate that growth of ERMS showing canonical Hh signaling activity due to *Ptch* germline mutations is efficiently reduced by SMO antagonists. This goes along with strong downregulation of the Hh target *Gli1*. Likewise *Ptch* mutant tumors are highly responsive toward the PI3K inhibitor pictilisib, which involves modulation of AKT and caspase activity. Pictilisib also modulates Hh target gene expression, which, however, is rather not correlated with its antitumoral effects. In contrast, sporadic ERMS, which usually express HH target genes without having *PTCH* mutation, apparently lack canonical HH signaling activity. Thus, stimulation by Sonic HE (SHH) or SAG (Smoothened agonist) or inhibition by SMO antagonists do not modulate HH target gene expression. In addition, SMO antagonists do not provoke efficient anticancer effects and rather exert off-target effects. In contrast, pictilisib and other PI3K/AKT/mTOR inhibitors potently inhibit cellular growth. They also efficiently inhibit HH target gene expression. However, of whether this is correlated with their antitumoral effects it is not clear. Together, these data suggest that PI3K inhibitors are a good and reliable therapeutic option for all ERMS, whereas SMO inhibitors might only be beneficial for ERMS driven by *PTCH* mutations.

## Introduction

Hedgehog (HH) signaling plays a major role in a variety of human cancers. The main components of the canonical HH signaling pathway are HH ligands, the transmembrane proteins Patched (PTCH) and Smoothened (SMO) and GLI transcription factors. In the absence of the HH ligand, PTCH represses SMO. Upon binding of HH to PTCH this repression is released. This leads to accumulation of SMO in the primary cilium where it triggers the modulation of a variety of proteins, which finally results in translocation of the GLI2 and GLI3 transcription factors into the nucleus. This activates the expression of HH target genes, which include GLI1 that can amplify the response on transcriptional level. Indeed, *GLI1* transcripts serve as the most reliable readout for active HH signaling. Alternative HH-target genes are *HHIP* and *PTCH*. However, in contrast to *GLI1*, the prediction of pathway activity from *HHIP* and *PTCH* levels is more difficult, because the respective proteins mediate a negative feedback by sequestering HH [for review see (1)].

The first link between HH signaling and cancer was the discovery of mutations in the HH receptor PTCH in the rare autosomal dominant inherited basal cell nevus syndrome (BCNS, also known as NBCCS or Gorlin Syndrome). Almost all BCNS patients develop basal cell carcinoma (BCC) that show high *GLI1* expression and thus an active HH signaling cascade. In addition to BCC these patients are predisposed to other tumors, first of all to medulloblastoma (MB) and less frequently to rhabdomyoma and rhabdomyosarcoma (RMS) (1, 2). Therefore *PTCH* mutations are considered as driver mutations for these tumors. This is similar to heterozygous *Ptch*^+/−^ mice, which develop BCC, MB, and RMS at a high frequency (3–5).

Today it is known that HH signaling is also active in many sporadic tumors. Whereas activation of this pathway by somatic mutations occurs only in some tumors (e.g., BCC and MB), other tumors show overexpression of HH ligands, which is seen e.g. in pancreatic, lung, and prostate cancers [for review see (6)]. In addition, the activity of GLI transcription factors and the expression of HH target genes can also be regulated in a non-canonical manner independently of HH, PTCH, and SMO, which can occur e.g., in melanoma and astrocytoma [for review see (7)].

Recently, we and others showed that sporadic RMS express HH ligands and the HH targets *GLI1* and *PTCH* (8–10). This led to the hypothesis that the HH pathway in sporadic RMS may be activated in a canonical ligand-dependent manner.

RMS is the most common pediatric soft tissue sarcoma with poor prognosis for high-risk patients (11). In children, RMS is divided by histology in embryonal (ERMS) and alveolar RMS (ARMS). The latter one can be classified by PAX/FOXO1-gene fusion status as fusion-positive or fusion-negative. Fusion-positive ARMS are very aggressive, whereas fusion-negative ARMS are clinically and molecularly indistinguishable from the less aggressive ERMS subtype (12). Interestingly, ERMS and fusion-negative ARMS exhibit significantly higher HH target gene expression compared to fusion-positive ARMS (8, 9).

Because HH target gene expression in sporadic RMS may be driven by HH ligands i.e., via the canonical HH/PTCH/SMO axis, we recently tested SMO inhibitors including vismodegib and sonidegib for their antitumorigenic effects in human cell lines derived from sporadic RMS. However, the effects of the drugs and the response of the cells were very heterogeneous and did not necessarily correlate with inhibition of HH signaling. Thus, some drugs paradoxically induced cellular proliferation at certain concentrations or showed antiproliferative effects without reduction of HH signaling activity (13). Therefore we hypothesize that (i) reliable anticancer effects using SMO inhibitors may only be achieved in RMS cells harboring *PTCH* mutations, (ii) some of the above-mentioned results may reflect off-target effects of the SMO inhibitors, and (iii) the HH pathway in RMS is not only regulated via the canonical axis, but also in a HH/PTCH/SMO-independent manner e.g., by the PI3K/AKT/mTOR cascade. The latter assumption is based on the observation that the PI3K/AKT axis plays a crucial role in RMS (14–17) and, like in many other tumors or tumor cell lines [e.g., (18–20)], shows cooperation with HH signaling in RMS cells (21).

We here tested these 3 assumptions by analyzing ERMS cells harboring a *Ptch* mutation and ERMS cells without obvious *PTCH* mutations. We used the SMO inhibitors vismodegib, sonidegib (both have been approved for advanced BCC), or HhAntag alone or in combination with PI3K/AKT/mTOR-inhibitors (pictilisib, PI-103, MK-2206, rapamycin, or everolimus) and analyzed the impact on the HH and PI3K pathway and on growth behavior of ERMS cells.

## Materials and methods

### Drugs

All used drugs, the respective provider, solvents, applications, and final concentrations are listed in Supplementary Table S1.

### Cell culture

The human ERMS cell lines RD and RUCH-2 were obtained from ATCC [for cell lines see (22)] and were cultured in DMEM, 10% FCS (Thermo Fisher Scientific Inc., Waltham, MA, United States of America) and 1% penicillin/streptomycin (PAN Biotech GmbH, Aidenbach, Germany; stock 10.000 U/ml).

SHH conditioned media (SHH-CM) or respective control-conditioned media (control-CM) were produced from HEK293 cells stably transfected with a SHH expression plasmid or from non-transfected HEK293, respectively, as described by Chen et al. (23). Shh-responsive B9 cells served as positive controls (24).

For preparation of primary murine RMS cultures the tumor was chopped with a razor blade and incubated with 3 mg/ml collagenase H (Roche, Mannheim, Germany) in DMEM for 60 min at 37°C while shaking with 1000 rpm. To release remaining cell aggregates the suspension was strained through a 40 μm nylon filter. Cells were sedimented at 300x g, 4°C for 5 min. The pellet was resuspended in DMEM containing 10% FCS and 1% penicillin/streptomycin and cells were seeded on collagen coated 96-well plates for proliferation analysis or 12-well plates for qRT-PCR analysis and western blot analysis. When 90% of cells were adherent, experiments were started. Cells were kept in culture for no longer than 4 days.

For analysis of cilia NIH/3T3 cells (control cells), RD and RUCH-2 were seeded at a density of 20.000 cells (standard cell culture condition) or 250,000 cells (highly confluent), respectively, per well of a 4 chamber CultureSlide (Falcon) and cultured for 48 h in 10% FCS (standard conditions). In another set of experiments the medium was replaced after 24 h by starvation medium (0.5% FCS). Then the cells were stained with antibodies against acetylated α-tubulin (Sigma Aldrich, T6793, clone 6-11B-1; 1:500) and Alexa488-conjugated anti-mouse secondary antibody (Dianova, 715-545-150, 1:400). Cells were mounted with ProLong Gold antifade reagent with DAPI (Life Technologies) and analyzed by fluorescence microscopy on a confocal laser scanning microscope equipped with software Fluoview FV100 (Olympus Corporation).

For BrdU incorporation assay with human ERMS cell lines 5,000 cells/well were seeded in 96-well-plates. After 24 h, the cells were incubated for 24 h with the respective drugs in the presence of BrdU. For BrdU incorporation and cell viability (WST-1) assay with the slow growing primary murine ERMS cells, cultures were grown to 90–100% confluency and were treated for 24 h with the respective drugs and for another 24 h with the respective drugs in the presence of BrdU. BrdU incorporation was measured using Cell Proliferation BrdU ELISA (Roche Diagnostics GmbH, Mannheim, Germany). The data are presented as the percentage of BrdU incorporation measured in time-matched solvent-treated controls that were set to 100%. Combination indices were calculated by CompuSyn software (25). Values >100% were not considered for calculation. Drug treatment was also 12 or 48 h for the cell viability assay. Afterwards WST-1 (Roche Diagnostics GmbH, Mannheim) was added to fresh culture medium and cells were incubated with this medium for 4 h. The colorimetric reaction was measured in a microplate reader at a wavelength of 450 nm.

For determination of apoptosis 100,000 RD cells/well were seeded in 6-well-plates. After treatment for 48 h with medium supplemented with drugs or solvent as indicated in the respective experiments, cells were stained with Annexin V-FITC (BD Biosciences, Heidelberg, Germany) and propidium iodide (PI, Miltenyi Biotec, Bergisch Gladbach, Germany) and apoptosis was determined by flow cytometry on a FACSCalibur (BD Biosciences, Heidelberg, Germany) equipped with FlowJo software (Tree Star Inc., Ashland, Oregon, United States of America).

If not stated otherwise, data shown summarize three independent experiments performed as triplicates.

### Real-time quantitative RT-PCR-analyses

For gene expression analysis 100,000 cells/well were seeded in 6-well-plates and allowed to attach for 24 h. After subsequent incubation of the cells for 24 h total RNA was isolated using TRIzol Reagent (Invitrogen GmbH, Karlsruhe, Germany). For RNA isolation from tissue samples, approximately 25 mg of tissue was chopped and homogenized on ice. RNA was isolated using TRIzol Reagent. cDNA was synthesized using Superscript II and random hexamers (Invitrogen, Karlsruhe, Germany). Gene expression was analyzed on the ABI Prism HT 7900 Detection System instrument and software (Thermo Fisher Scientific Inc., Waltham, United States of America) using SYBR-green-based qRT-PCR assays. Relative quantification was done by the standard curve method. All primer pairs except for *18S rRNA* primers were intron-flanking and are shown in Supplementary Table S2.

Amplification of *18S rRNA* served to normalize the amount of sample cDNA. Graphs represent mean value of three independent experiments measured in triplicates plus standard error of the mean (s.e.).

### Immunohistochemistry

Isolated tumor samples were embedded in paraffin and sectioned for histological analyses. The identity of RMS was confirmed by examination of Haematoxylin and Eosin (H&E) stained sections. Paraffin sections were stained using the primary and secondary antibodies described in Supplementary Table S3. Supplementary Table S3 also gives the respective dilutions and the respective antigen retrieval methods. Diaminobenzidine chromogen was used as substrate.

### Western blot analysis

Cells were lysed in RIPA buffer containing 50 mM Tris-HCl (pH 7.4), 150 mM NaCl, 1 mM EDTA, 1% NP-40, 0.25% Na-deoxycholate, and a protease inhibitor cocktail (Merck KGaA, Darmstadt, Germany). For protein isolation from tissue samples, approximately 30 mg tissue was chopped and homogenized on ice. Protein concentrations were determined by the Pierce Protein BCA Assay Kit (Thermo Fisher Scientific Inc., Waltham, United States of America). Primary antibodies used to detect the individual target proteins and corresponding secondary antibodies are shown in Supplementary Table S3. Pierce ECL Plus Western Blot substrate (Thermo Fisher Scientific Inc., Waltham, United States of America) and the FluorChem Q detection system (Bio-Techne Corp., Minneapolis, United States of America) were used for visualization of protein signals. All Western blots shown are representative for at least two independent experiments.

### Breeding of RMS-bearing mice, drug treatment, and tumor measurements

*Ptch*^*del*/+^ mice on a C57BL/6N (B6) background, which harbor a heterozygous deletion of exon 8 and 9 within the *Ptch* gene (see Zibat et al. (26) for generation of *Ptch*^*del*/+^ from *Ptch*^*flox*/*flox*^ mice) were bred to Balb/c (Balb) wildtype (wt) mice to obtain a mixed B6/Balb genetic background that confers high susceptibility for RMS (27). The resulting B6xBalb*-Ptch*^*del*/+^ mice are named *Ptch*^+/−^ mice. Genotyping of the mice was done as described (26) (primers are additionally shown in Supplementary Table S2). RMS of *Ptch*^+/−^ mice were detected upon weekly manual palpation.

RMS-bearing *Ptch*^+/−^ mice were treated twice a day orally with vehicle/vehicle (controls), single drug/vehicle or drug/drug combinations as indicated in the respective experiments. Between the daily treatments we left an interjacent time span of 5 h to avoid potential drug-drug interactions or complexations, which could have lowered the intestinal resorption and hence the therapeutic efficacy of the combination treatment. The preparation of the drug suspensions and the dosing are given in Supplementary Table S1. The same volume of the vehicle methyl cellulose tween (MCT) was given to control mice.

Animals that received HhAntag and/or pictilisib were treated for a period of 35 days. Tumor size was monitored by low dose *in vivo* microCT before therapy onset, at day 21 and at the end of treatment. Since the tumors after combination treatment were too small to perform all molecular analyses, the treatment period was reduced to 14 days and tumors were isolated without subjection of the animals to microCT. In addition, the treatment period for vismodegib and sonidegib in combination with pictilisib was reduced to 21 days. The tumor size in these studies was monitored by microCT before therapy onset and at the end of treatment. In all settings, tumors for molecular analysis were isolated 1–4 h after the last treatment, which was either pictilisib or vehicle.

For microCT mice were anesthetized with 1–2% isoflurane in 1:1 air:oxygen mix. In order to visualize the tumors 5 ml/kg of contrast agent Imeron 300 was injected into the tail vein approximately 30 sec prior imaging. The Quantum FX MicroCT (Perkin Elmer, Waltham, United States of America) was used for *in vivo* imaging of tumor bearing mice at 90 kVp tube voltage, 200 μA tube current and a 2 min total acquisition time. Data sets were reconstructed with a voxel size of 80 μm and analyzed using Scry v6.0 software (Kuchel & Sautter UG, Bad Teinach-Zavelstein, Germany).

All experiments using animals were performed in agreement with all relevant legal and ethical requirements and have been approved by the Lower Saxony State Office for Consumer Protection and Food Safety (file number 33.14-42502-04-13/1084).

### Statistical analysis

*In vivo* tumor growth was either considered as logarithmic tumor volume or classified into progressive disease (PD), stable disease (SD), or partial response (PR) according to the RECIST criteria (treatment response). The logarithmic tumor volume was considered for the analysis relative to the start of the treatment to avoid inter-mice variation. Treatment response was compared between treatment regimens or combinations thereof by two-sided Fisher's exact test. Relative logarithmic tumor volume was modeled in random effects mixed models with repeated volume measurements, adjusted for age at the start of treatment and sex. Multiple tumors of a mouse were considered as independent observations. The variance of tumor volume was allowed to vary between treatment regimes.

Multiple testing was addressed according to the method of Dunnett (differences between one control and several active treatment regimens) or Tukey-Kramer (differences among all treatment regimens), as appropriate. Spearman correlation was estimated between relative *Gli1* expression and relative tumor size, and therefore being independent of scaling of these quantities.

Results are plotted as mean values plus one s.e. if not stated otherwise. The level of significance was set to α = 0.05. We used SAS 9.4 and GraphPad Prism 6 software to perform all statistical analyses.

## Results

### SMO or PI3K inhibitors inhibit *Gli1* expression in cultured *Ptch* mutant ERMS cells

First, we analyzed the effectiveness of the SMO inhibitors vismodegib, sonidegib, or HhAntag and of the pure PI3K inhibitor pictilisib (also known as GDC-0941; used in the clinics) in *PTCH* mutant ERMS cells. *PTCH* mutations are driving RMS in BCNS patients whereas they are very rare in sporadic human RMS (2, 28–30). Since a siRNA-mediated knock-down of *PTCH* in a cell line derived from a sporadic ERMS would probably not adequately reflect this situation (i.e., it would not be a driver mutation), we tested the drugs in primary tumor cell cultures from *Ptch* mutant mice (*Ptch*^+/−^). As humans with *PTCH* mutations these mice develop ERMS-like tumors that show active HH signaling. In addition they show increased Akt activity (4, 31).

**Figure 1:**
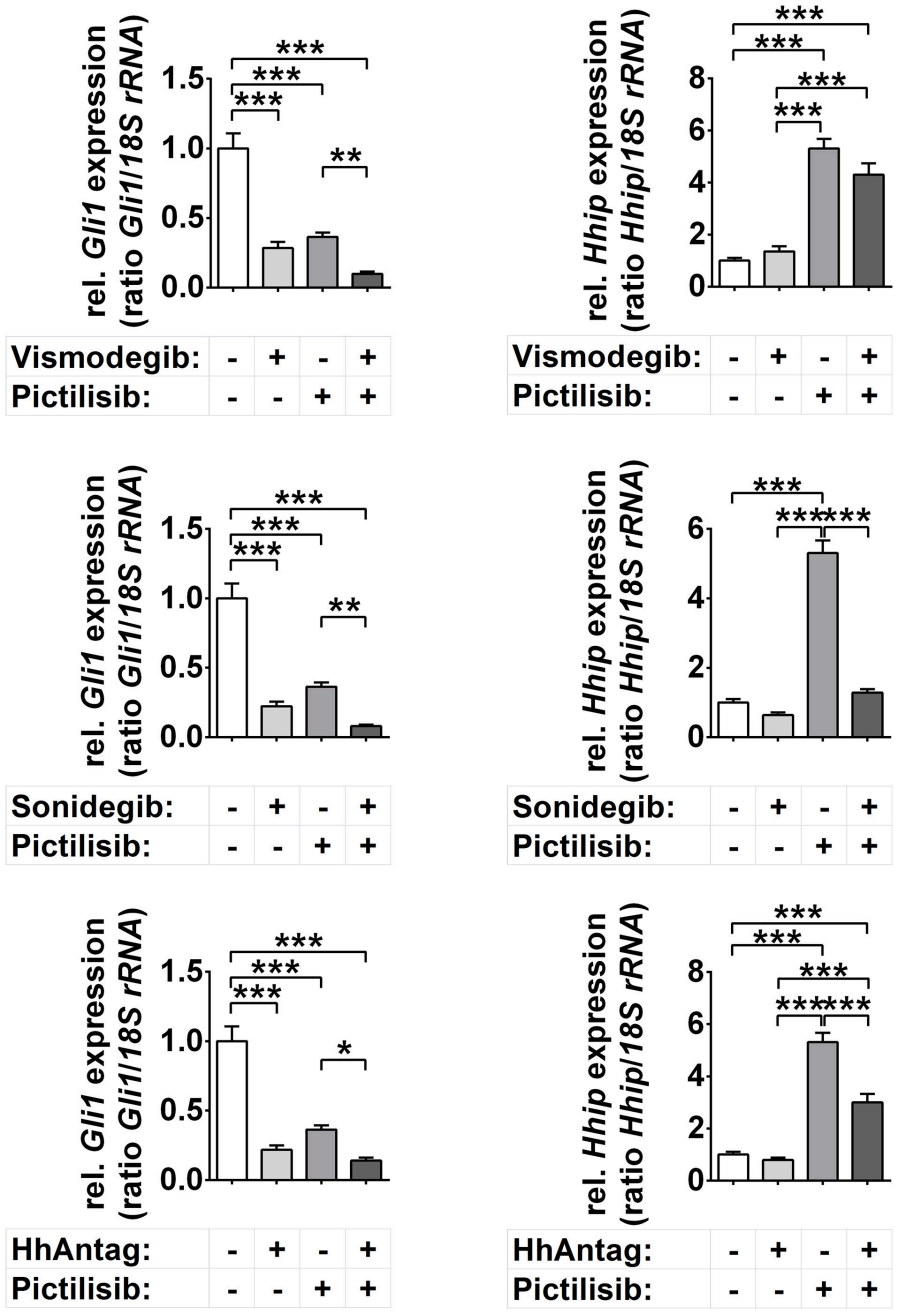
Effects of Smo and/or Pi3k inhibition on Hh target gene expression. *Gli1* and *Hhip* expression in cells treated for 48 h with 10 μM vismodegib, 10 μM sonidegib, 10 μM HhAntag or 5 μM pictilisib. Bars represent the mean +s.e. of three independent experiments measured in duplicates. ^*^P < 0.05, ^**^P < 0.01, ^***^P < 0.001 when analyzed by Tukey's test for multiple comparisons.

In general, the incubation of the primary murine tumor cells with vismodegib, sonidegib, or HhAntag significantly reduced the expression of the Hh target *Gli1*, but not of *Hhip* (Figure 1). Treatment with the pure PI3K inhibitor pictilisib (also known as GDC-0941; used in the clinics) also significantly reduced *Gli1* levels (Figure 1), but at the same time significantly increased the expression of *Hhip*. Moreover, combined SMO inhibitor/pictilisib treatment resulted in a stronger downregulation of *Gli1* than single treatments though it did not reach significance. Finally, the drugs sonidegib and HhAntag reversed the pictilisib-mediated upregulation of *Hhip* to basal levels (Figure 1).

The unchanged *Hhip* levels upon incubation with SMO inhibitors and the pictilisib mediated up- and downregulation of *Hhip* and *Gli1*, respectively, seem at the first glance puzzling. However, it is possible that *Hhip* is silenced in *Ptch*^+/−^ ERMS by hypermethylation as described for other HH-associated cancer types (32, 33). In this case and because active PI3K/AKT signaling stabilizes DNA methyltransferase 1 (Dnmt1) (34), pictilisib may have decreased the level of Dnmt1, resulting in *Hhip* upregulation. Hypermethylation of the *Hhip* promotor also could explain why SMO inhibitors did not affect *Hhip* expression levels although they were in general functional. However, this is pure speculation and remains to be analyzed in the future. Together, these data show that vismodegib, sonidegib, HhAntag, and also pictilisib downregulate the expression of the major Hh target gene *Gli1*, whereas the expression of the Hh target *Hhip* is unchanged (SMO inhibitors) or upregulated (pictilisib).

Western blot analysis furthermore showed that pictilisib, but none of the SMO inhibitors, efficiently suppressed phosphorylation of Akt, which is the key component of the PI3K/AKT/mTOR pathway. Pictilisib also downregulated the total Akt level and enhanced caspase 3 cleavage in *Ptch* mutant ERMS cells in a concentration-dependent manner (Supplementary Figure S1A). These pictilisib-mediated changes were not affected by SMO inhibitors (Supplementary Figures S1A,B).

SMO and/or PI3K inhibitor treatment also resulted in significantly reduced BrdU incorporation of *Ptch* mutant ERMS cells after 48 h (data not shown). However, a cell viability assay revealed that the treatments became toxic for the cells with time (Supplementary Figure S2).

Together we can conclude that both SMO and PI3K inhibitors are involved in regulation of Hh target genes, at least of *Gli1*, in this murine ERMS model.

### Strong anticancer effects of SMO or PI3K inhibitors in erms-bearing *Ptch* mutant mice

To evaluate the anticancer effects of SMO inhibitors and pictilisib *in vivo*, we treated ERMS-bearing *Ptch* mutant mice with vismodegib, sonidegib, HhAntag and/or pictilisib daily for 21 days (vismodegib and sonidegib cohorts) or 35 days (HhAntag cohort). All drugs were very well tolerated by the animals. Tumor growth was monitored by low dose *in vivo* microCT. For molecular analysis all tumors were isolated 1–4 h after the last vehicle or pictilisib treatment (see Materials and Methods section).

**Figure 2:**
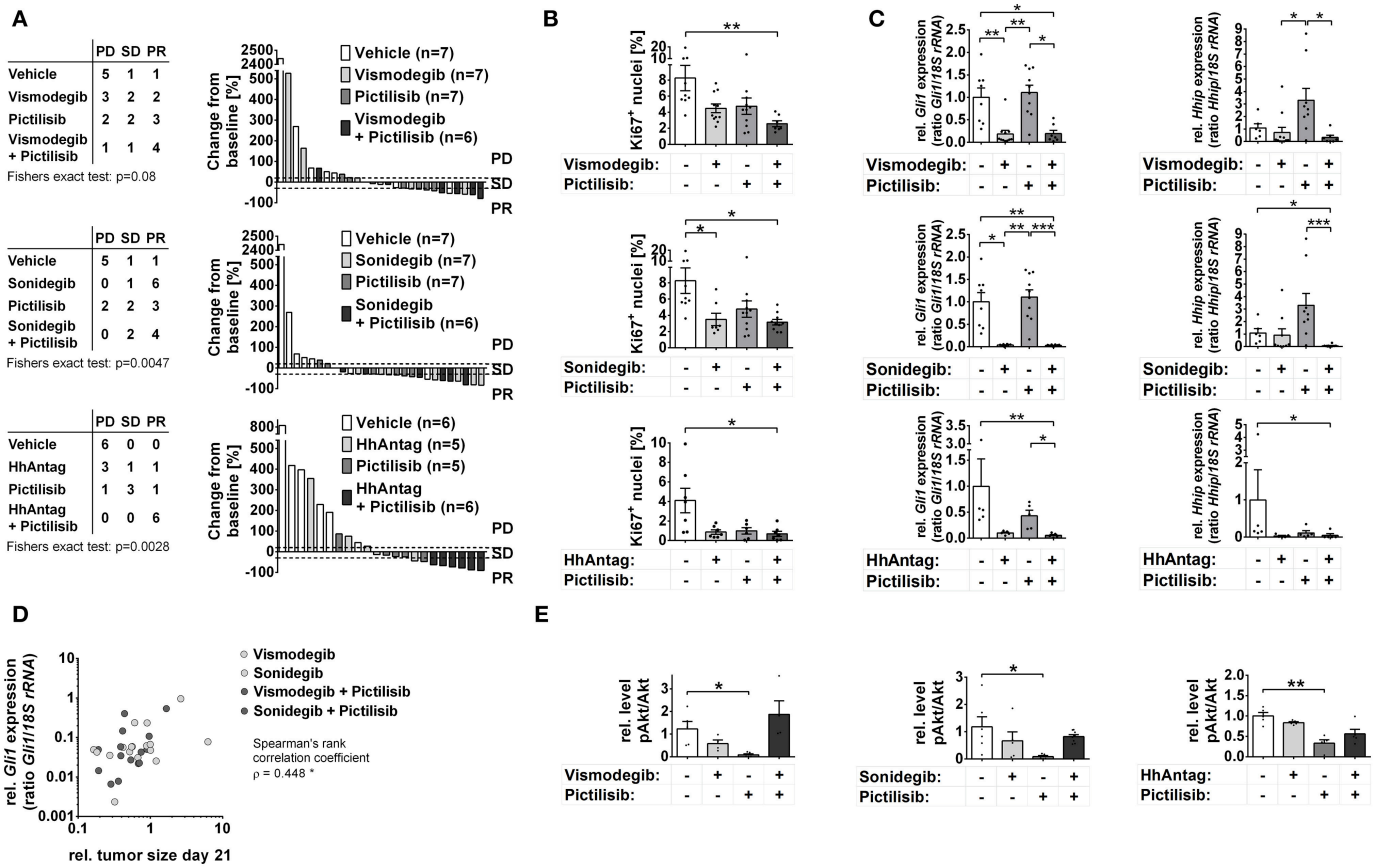
Effects of Smo and/or Pi3k inhibition on *in vivo* growth of *Ptch* mutant ERMS. Analysis of RMS of *Ptch*^+/−^ mice that have been treated orally for 21 (vismodegib and/or pictilisib *n* = 27; sonidegib and/or pictilisib *n* = 27) or 35 days (HhAntag and/or pictilisib *n* = 22) as indicated. **(A)** Disease progression was classified according to RECIST criteria as progressive disease (PD), stable disease (PD) or partial response (PR). Right panel shows individual changes in tumor growth as measured by microCT at therapy end in comparison to the tumor size at therapy onset. **(B)** Quantification of Ki67 positive cells of tumors shown in **A)**. **(C)** *Gli1* and *Hhip* expression in tumor samples collected at day 21 (vismodegib and/or pictilisib study or sonidegib and/or pictilisib study) or day 14 (HhAntag and/or pictilisib study; see main text for explanation). **(D)** Correlation of tumor growth changes that have been treated with SMO inhibitors with *Gli1* expression. **(E)** pAkt normalized to Akt expression levels as measured by semiquantitative densitometry of Western blot (Western blots and pAkt and Akt expression levels are shown in Supplementary Figures S3C,D, respectively). ^*^ *P* < 0.05, ^**^P < 0.01, ^***^P < 0.001 when analyzed by Dunn's test for multiple comparisons.

The *in vivo* tumor growth analysis revealed an increase in mice with stable disease (SD) or partial response (PR) by mono-drug treatment with vismodegib, sonidegib, HhAntag, or pictilisib (*p* = 0.0003 considering any mono-drug treatment; *p* = 0.0152 for pictilisib and *p* = 0.0210 for sonidegib) (Figure 2A). Sonidegib mono-treatment led to PR in the majority of mice similar to the combination treatments sonidegib/pictilisib or vismodegib/pictilisib. However the most effective therapy seems to be the combination treatment HhAntag/pictilisib, since all mice reached a partial treatment response (Figure 2A). However, this ranking is done regardless to statistical uncertainty. Furthermore, animals treated with HhAntag/pictilisib have received the drugs for 35 days, whereas mice of the vismodegib/pictilisib and sonidegib/pictilisib study have been treated for 21 days (see material and methods). To mirror the duration of treatment, we fitted mixed random effects models to the growth in tumor volume (Supplementary Figure S3A; see material and methods). For a mono-drug treatment with vismodegib or HhAntag one can expect no essential tumor growth, since the tumor volume at the end of therapy was unchanged [for e.g., vismodegib relative tumor volume (rel.tv) = 0.96 95%-confidence interval (conf.I) [0.56–1.65]]. On the other hand sonidegib or pictilisib are expected to induce tumor regression, since the tumor volume was reduced at the end of therapy compared to vehicle treatment [for e.g., sonidegib rel.tv = 0.32 95%-conf.I [0.15–0.68]]. When vismodegib or sonidegib are combined with pictilisib tumor regression will be similar as for single pictilisib treatment [for e.g., sonidegib/pictilisib rel.tv = 0.49 95%-conf.I [0.26–0.94]], whereas the combination HhAntag/pictilisib will result in tumor regression that is more efficient than the single treatments (Supplementary Figure S3A).

In summary, these data demonstrate that *in vivo* treatment of *Ptch* mutant ERMS with SMO inhibitors and/or pictilisib either stop or reduce tumor growth and that sonidegib monotherapy or a HhAntag/pictilisib combination therapy show the strongest antitumor effects.

To substantiate these findings the proliferative activity of the tumors was analyzed by Ki67 immunhistochemical stainings. In agreement with the tumor growth analyses, all single and combination treatments strongly reduced the number of proliferating cells compared to vehicle-treated samples (Figure 2B). The reduction became significant in tumors that have been treated with sonidegib alone or with all of the drug combinations (Figure 2B).

### Suppression of *Gli1* expression correlates with size reduction of *Ptch* mutant erms upon treatment with SMO inhibitors, but not with PI3K inhibitors

Analyses of Hh signaling activity revealed extremely reduced *Gli1* expression levels after treatment with SMO antagonists, whereas the expression of *Hhip* was unaltered. This is identical to the cell culture data. However, in contrast to the *in vitro* experiments pictilisib monotherapy did not inhibit *Gli1* transcription in the tumors, whereas the pictilisib-mediated upregulation of *Hhip* was also seen *in vivo* (Figure 2C; please note the missing pictilisib-mediated upregulation in the HhAntag cohort. However, in order obtain enough material for RNA analysis, the tumors of this cohort have only been treated for 14 days whereas the others have been treated for 21 days. Therefore it is possible that pictilisib-mediated activation of *Hhip* expression requires more than 14 days).

Correlation analyses of *Gli1* expression levels (see Figure 2C) and ERMS size (see Figure 2A) corroborates these results. Whereas *Gli1* expression did not correlate with tumor size in the vehicle- or pictilisib-treated groups (Supplementary Figure S3B), a significant correlation was observed in the groups that have been treated with vismodegib, sonidegib, vismodegib/pictilisib, or sonidegib/pictilisib (Figure 2D; data was not available for HhAntag, see Material and Methods). Together these data show that the antitumor effects of SMO inhibitors in *Ptch* mutant ERMS are correlated with a decrease in *Gli1* levels, whereas the antitumor effects of pictilisib are not.

In order to investigate whether antitumoral efficacy of pictilisib was rather associated with inhibition of Pi3k signaling, the Akt phosphorylation status and caspase 3 cleavage was investigated in the tumors via Western blot analyses. Unfortunately, caspase 3 cleavage was very hard to detect in the samples. However, pictilisib-treated tumors showed a downregulation of pAkt levels in comparison to the tumors that have been treated with SMO inhibitors (Figure 2E; Supplementary Figures S3C,D). Surprisingly, when pictilisib was combined with SMO inhibitors this effect was abrogated (Figure 2E;Supplementary Figures S3C,D).

In summary, these results demonstrate that SMO antagonists can efficiently block growth or even induce regression of *Ptch* mutant ERMS, which goes along with efficient reduction of *Gli1* expression. Therefore, SMO antagonists seem to be appropriate anticancer drugs for RMS that show canonical Hh signaling activity due to *Ptch* mutations. Similarly, the PI3K inhibitor pictilisib stops growth of *Ptch* mutant tumors, which is accompanied by inhibition of Akt phosphorylation and most likely by downstream processes such as induction of apoptosis, but not by inhibition of Hh target genes.

### PI3K/AKT/mTOR-mediated activation of HH target gene expression in *Ptch* wt ERMS cells

As demonstrated above, the antitumoral response of individual ERMS driven by *Ptch* mutations toward different SMO antagonists is rather homogeneous. This is different from cell lines derived from human sporadic ERMS, which are most likely all wt for *PTCH* and which show a highly diverse response to SMO antagonists (see introduction). Since human ERMS—regardless of the general lack of *PTCH* mutations (i.e., *PTCH* mutations in sporadic RMS are exceedingly rare)—show high *GLI1* expression (8) and express HH ligands (10), we hypothesized that HH signaling activity in ERMS is caused in a ligand-dependent manner. However, when we tried to stimulate HH signaling activity in the ERMS cell lines RD, that is *PTCH* wt (35), or RUCH-2, neither SHH-conditioned medium (SHH-CM) nor the SMO agonist SAG uniquely induced the expression of *GLI1, HHIP* or *PTCH* (Supplementary Figures S4A–C). As the cells did not develop cilia under the respective experimental conditions (Supplementary Figure 4D), the results implicate that canonical HH signaling cannot be activated in ERMS cells that are wt for *PTCH*.

Since *GLI1* and *HHIP* expression can be modulated by PI3K inhibitors in *Ptch* mutant ERMS cells (see above), we next incubated the human RMS cell lines RD and RUCH-2 with pictilisib. We also employed the dual PI3K/mTOR inhibitor PI-103, the pure AKT inhibitor MK-2206 or the mTOR inhibitors everolimus and rapamycin. These drugs were used alone or in combination with vismodegib, sonidegib or HhAntag.

**Figure 3:**
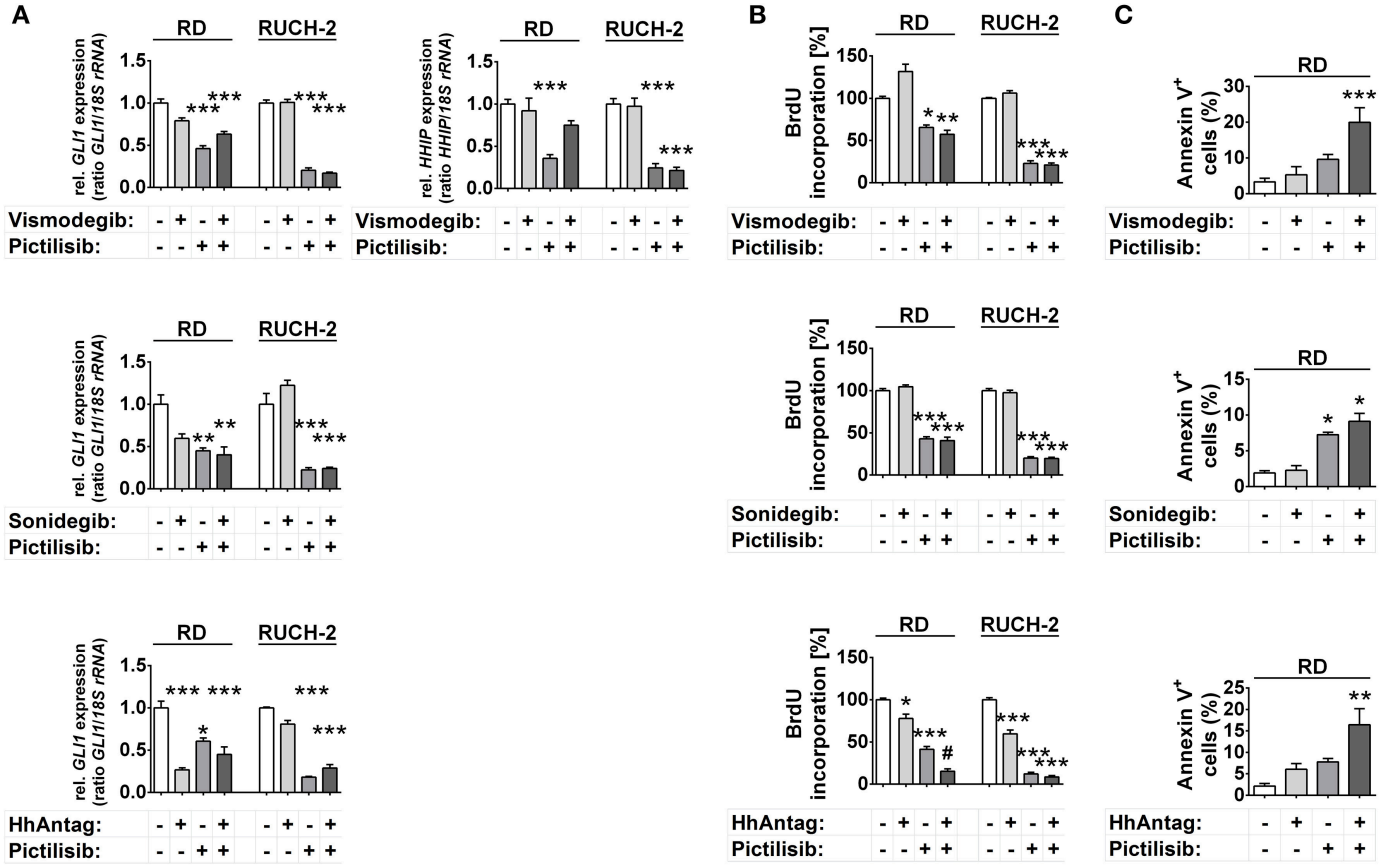
Effects of SMO and/or PI3K inhibition on HH target gene expression, proliferation and apoptosis in human ERMS cell lines. RD and RUCH-2 cells treated with 10 μM of SMO inhibitors and/or 10 μM of the PI3K inhibitor pictilisib. **(A)** *GLI1* expression levels after treatment for 24 h. *HHIP* expression was investigated after treatment with vismodegib and/or pictilisib **(B)** BrdU incorporation after treatment for 24 h. BrdU incorporation of solvent treated cells was set to 100%. Bars represent the mean + s.e. of three independent experiments performed in triplicates. **(C)** Annexin V staining and subsequent FACS analysis of RD cells treated for 48 h with the drugs as indicated. Bars represent the mean number of Annexin V^+^ cells + s.e. of two independent experiments performed in duplicates.^*^P < 0.05, ^**^P < 0.01, ^***^P < 0.01 compared to cells treated with solvent and analyzed by Tukey's test for multiple comparisons. ^#^*P* < 0.05 compared to cells treated with either drug alone.

Analyses of the *GLI1* transcription levels revealed that only HhAntag, but not vismodegib or sonidegib, significantly inhibited *GLI1* expression in RD cells (Figure 3A). However, HhAntag did not alter *GLI1* expression in RUCH-2 cells (Figure 3A). In addition, *HHIP* levels were not affected by SMO inhibitors (at least not by Vismodegib; Figure 3A). In contrast, pictilisib efficiently inhibited *GLI1* and *HHIP* expression in both cell lines (Figure 3A). This was similar for other PI3K, AKT and/or mTOR inhibitors, which potently inhibited *GLI1* expression in both cell lines (Supplementary Figure S5A). We also combined the PI3K, AKT and/or mTOR inhibitors and SMO inhibitors to search for potential cooperative effects in inhibition of *GLI1* expression. However, none of the combinations was able to significantly intensify the inhibition (Figure 3A, Supplementary Figure S5A).

Together the data suggest that HH target gene expression in human ERMS cells is regulated by PI3K signaling and occurs downstream of PI3K at the level of AKT or mTOR.

### HhAntag and the PI3K inhibitor pictilisib, but not vismodegib or sonidegib, can induce anticancer effects in *Ptch* wt ERMS cells

We next investigated the antitumoral potential of pictilisib and the other PI3K/AKT/mTOR inhibitors compared with that of SMO inhibitors. For this purpose we measured proliferation and apoptosis of RD and/or RUCH-2 cells after inhibitor treatment.

HhAntag and pictilisib and also PI-103 decreased proliferation of RD cells, whereas sonidegib had no effect and vismodegib revealed the already described paradoxical pro-proliferative effect (Figure 3B, Supplementary Figure S5B) (13). Similarly HhAntag, pictilisib, PI-103 and also MK-2206 had antiproliferative effects in RUCH-2 cells, whereas sonidegib and vismodegib had not (Figure 3B, Supplementary Figure S5B). Furthermore, a cooperative antiproliferative effect was induced in RD cells by the combination HhAntag (but not vismodegib or sonidegib) and pictilisib (Figure 3B). In RUCH-2 cells cooperative effects were detected by the combinations sonidegib/rapamycin, HhAntag/PI-103, HhAntag/MK-2206, and HhAntag/rapamycin (Supplementary Figure S5B).

Measurement of apoptotic RD cells showed a moderate increase of apoptosis after pictilisib treatment, but not after treatment with SMO inhibitors (Figure 3C). Similarly, PI-103 induced apoptosis. However, MK-2206, everolimus and rapamycin had no effect (Figure 3C, Supplementary Figure S5C). The combination treatments with vismodegib/pictilisib, HhAntag/pictilisib (Figure 3C) or HhAntag/PI-103 (Supplementary Figure S5C) enhanced the apoptosis rate, however it was not a cooperative effect (please again note that combination treatments were considered cooperative when data were significant compared to either single treatment and to control). A significant cooperative proapoptotic effect was observed for HhAntag/MK-2206 (up to 45%; Supplementary Figure S5C). Apoptosis measurement of RUCH-2 cells was not possible due to the strong adherence of the cells to the culture dish, which would require aggressive trypsinization of the cells leading to unreliable apoptotic assay results.

To evaluate if all drugs were functional and the observed antitumoral effects were due to differences in the activation status of proteins involved in the PI3K/AKT/mTOR axis, caspase 3 cleavage, the phosphorylation status of AKT and of the mTOR downstream target S6 were investigated. The results are shown and described in detail in Supplementary Figure S6. In short, all PI3K/AKT/mTOR inhibitors were functional as shown by downregulation of pAKT and pS6 levels. As already described by our lab (13), HhAntag reduced pAKT in RD and RUCH-2 cells. We also found that vismodegib and sonidegib, but not HhAntag, enhanced the total level of AKT in RUCH-2 cells, but not in RD cells.

HhAntag significantly inhibited cellular proliferation of both RD and RUCH-2 cells. This went along with significant inhibition of HH signaling in RD but not in RUCH-2 cells (compare Figures 3A–C). Therefore it is tempting to speculate that the reduced pAKT levels, and not GLI1 levels, are responsible for the antiproliferative effects of HhAntag in comparison to vismodegib or sonidegib. However, this is pure speculation and remains to be elucidated in the future. Contrarily, a more obvious correlation is the enhanced cleavage of caspase 3 and the significant cooperative proapoptotic effect in RD cells that is seen upon treatment with HhAntag/MK-2206.

In summary, SMO antagonists do not show strong antitumoral effects in cultured *PTCH* wt ERMS cells. Indeed, their efficacy in terms of inhibition of proliferation or induction of apoptosis is much weaker compared to PI3K/AKT inhibitors. Finally, the results suggest off-target effects of SMO antagonists, because they can act on other signaling pathways (e.g., on AKT signaling).

## Discussion

We here tested whether reliable anticancer effects using SMO inhibitors may only be achieved in RMS cells harboring *PTCH* mutations. Indeed, the three SMO inhibitors vismodegib, sonidegib, and HhAntag strongly downregulated *Gli1* expression in *Ptch* mutant ERMS cells both *in vitro* and *in vivo* and strongly induced antiproliferative effects *in vivo*. Furthermore, tumor growth inhibition correlated with *Gli1* expression levels, which suggests that *Gli1* is a perfect biomarker for the antitumor effects of SMO inhibitors in *Ptch* mutant ERMS.

This is different in ERMS without *PTCH* mutations (RD cells) or in which we were not able to activate HH target gene expression by SHH or SAG (RD and RUCH-2 cells). In addition, SMO inhibitors are rather ineffective with respect to inhibition of HH target expression and proliferation. This strongly argues against canonical HH signaling activity in these cells and is similar to other reports showing that SMO inhibitors are mainly effective in medulloblastoma of the SHH subgroup (36) and in basal cell carcinoma that most frequently are driven by *PTCH* mutations (37), whereas they are of no benefit in e.g. pancreatic cancer or lung cell cancer, in which SHH overexpression was thought to be responsible for HH signaling activity in the tumors [for review see (38, 39)]. Thus our data support the recent hypothesis that SMO inhibitors are effective only in tumors driven by mutations in the HH pathway, whereas they in general lack efficacy in other tumors^1^ (39). Nevertheless, there is a chance that these drugs might have worked in RD or RUCH-2 xenografts, because the tumor microenvironment plays an important role in canonical HH signaling activity [for review see (39)]. Indeed, a recent paper shows that SMO-deficient RD cells do rarely form palpable tumors. However, as stated by the authors, this effect is not due to changes in cell viability, cell cycle or proliferation, but to unsuccessful tumor initiation (40). Thus, it is rather unlikely that SMO inhibitors may induce stable disease or even regression of ERMS that are wt for *PTCH*.

We also tried to elucidate whether SMO inhibitors display off-target effects. When used in targeted therapy, vismodegib, sonidegib, and HhAntag only should hit SMO and thus should block activity of GLI transcription factors. However, vismodegib and sonidegib can increase total AKT level and HhAntag can decrease pAKT levels. Although this observation is not surprising [i.e., canonical HH activity is known to regulate AKT (41–43)], it strongly argues for off-target effects of the drugs. Indeed, off-target effects can occur when SMO inhibitors are used at high concentrations [for review see (39)]. Finally, whereas a drug-induced decrease of pAKT may be beneficial in tumor therapy, an increase of total AKT may be deleterious for therapy outcome (44).

In addition, our data show that *vice versa* PI3K/AKT/mTOR signaling regulates HH/Hh target gene expression in ERMS.

In *Ptch* mutant ERMS we found that pictilisib upregulated *Hhip* expression both *in vitro* and *in vivo*. We also found that pictilisib reduced *Gli1* levels in culture, but not when applied orally to the mouse. Whereas the pictilisib-mediated *Hhip* upregulation can be explained by epigenetic events (please see Results section), the different effects on *Gli1* expression in the *in vitro* vs. the *in vivo* situation could be due to e.g. drug pharmacokinetics or the tumor microenvironment. However, since pictilisib potently reduced growth of *Ptch* mutant tumors *in vivo* without modulation of *Gli1*, its strong antitumoral effects in *Ptch*^+/−^ mice are most likely not related to inhibition of Hh signaling.

In *Ptch* mutant ERMS, pictilisib also intensified antitumoral effects of some SMO antagonists. In this case, intensification may indeed rely on inhibition of HH signaling mediated by SMO inhibitors. However, it is apparently independent from the phosphorylation status of AKT. Thus, the pictilisib-mediated inhibition of AKT phosphorylation is abolished when pictilisib is applied in combination with SMO inhibitors.

Pictilisib and other PI3K/AKT/mTOR inhibitors also inhibited *GLI1* expression in *PTCH* wt RD cells. We also found that pictilisib downregulated *HHIP*. Because PI3K signaling regulates the activity of GLI transcription factors in many other tumor entities [e.g., (19, 20, 45, 46)], it is now tempting to speculate that ERMS follows a similar scenario, in which the HH pathway in ERMS is regulated in a non-canonical manner. However, it is possible that PI3K/AKT/mTOR signaling modulates the expression independently of HH pathway components.

Furthermore, the downregulation of HH target gene expression was accompanied by inhibition of cellular proliferation and apoptosis induction. Thus, it is possible that the antitumoral effects of PI3K/AKT/mTOR inhibitors in *PTCH* wt ERMS is partly mediated by HH target inhibition. However, the fact that MK-2206, everolimus or rapamycin reduce *GLI1* expression levels while not affecting cell proliferation or apoptosis, argues against this hypothesis.

Finally, a treatment of *PTCH* wt RD cells with the combinations vismodegib/pictilisib, HhAntag/pictilisib or HhAntag/PI-103 enhanced apoptosis. These results are similar to our recent data showing that concomitant inhibition of HH and PI3K/AKT/mTOR signaling using the GLI1/2 inhibitor GANT61 and the PI3K/mTOR inhibitor PI-103 synergistically induces apoptosis and growth reduction of cell lines derived from sporadic RMS. However, in contrast to the data shown here, this was associated with strong inhibition of *GLI1* expression and a cooperative effect on caspase-dependent apoptosis via the mitochondrial pathway (21). Indeed, GANT61 can attenuate the proliferation of both embryonal and alveolar RMS cells-derived xenograft tumors thereby blocking their growth (47). Since GANT61 blocks the HH pathway at the level of GLI these data again demonstrate the importance of these transcription factors in PTCH wt ERMS. Unfortunately, GANT61 is not very stable and therefore is currently not used in the clinics (48).

Together, this work highlights that the use of SMO antagonists in ERMS is a double-edged sword. Thus, our data suggest that these drugs are very effective in *PTCH* mutant ERMS that show canonical HH signaling activity, whereas they are in all likelihood ineffective in *PTCH* wt ERMS that show HH target gene expression due to activity of other signaling pathways. Therefore, treatment with these drugs requires patient selection by either pre-testing of SMO antagonists in patient-derived xenograft cultures or screening of patients for *PTCH* mutations.

In contrast, PI3K inhibition by e.g., pictilisib may be a superior and more general option in ERMS treatment. PI3K inhibition evokes strong and stable anticancer effects in both *PTCH* mutant and *PTCH* wt ERMS. Although pictilisib and other PI3K/AKT/mTOR inhibitors can modulate HH target gene expression, the role of this effect in ERMS response is not clear and remains to be established in the future.

## Author contributions

NG, RR, and HH contributed to conception and design of the study. JB, MK, KD, CD, and AU contributed to data acquisition and helped with data interpretation. AR performed the statistical analysis; H-US contributed to data interpretation. SF provided reagents and ideas; HH and NG wrote the manuscript. All authors read and approved the manuscript.

### Conflict of interest statement

The authors declare that the research was conducted in the absence of any commercial or financial relationships that could be construed as a potential conflict of interest.

## Footnotes

^1^https://am.asco.org/daily-news/hedgehog-signaling-pathway
