# Supplementary material for: Different Response of Ptch Mutant and Ptch Wildtype Rhabdomyosarcoma Toward SMO and PI3K Inhibitors
Source: Front Oncol. 2018 Sep 25;8:396. doi: 10.3389/fonc.2018.00396 (PMC6168716; doi:10.3389/fonc.2018.00396)
Supplement: Supplementary file 1 [file Data_Sheet_1.pdf]

frontiers

Supplementary Material

Different response of *Ptch* mutant and *Ptch* wildtype rhabdomyosarcoma towards SMO and PI3K inhibitors

Natalie Geyer, Rosalie Ridzewski, Julia Bauer, Maria Kuzyakova, Kai Dittmann, Christian Dullin, Albert Rosenberger, Hans-Ullrich Schildhaus, Anja Uhmann, Simone Fulda and Heidi Hahn*

*** Correspondence:** Heidi Hahn; hhahn@gwdg.de

1 Supplementary Figures and Tables

1.1 Supplementary Tables

Supplementary Table S1: Used drugs including provider, application, solvents and final concentrations. For *in vivo* treatment the drugs were dissolved in methyl cellulose tween (MCT) as recommended by the manufacturers. In short, 0.5 g methyl cellulose was solubilized in 40 ml hot double-distilled water (80-90°C). While stirring 40 ml cold H_2O were added and the suspension was refrigerated overnight. The next day, the clear solution was equilibrated to room temperature, 200 µl Tween-80 were added and the solution was filled up to 100 ml with H_2O. After sterile filtration the MCT solution was stored at 2-8°C for one month. MCT was used to solubilize the drugs, which were stored for a maximum of 24 h at 4°C. HhAntag was prepared directly before oral application.

**drug**	**provider**	**application**	**vehicle**	**conc.**
Cyclopamine	Sigma-Aldrich (St. Louis, USA)	*In vitro*	EtOH	5 – 10 µM
Vismodegib (GDC-0449)	Selleckchem (Munich, Germany)	*In vitro*<br>*In vivo*	DMSO<br>MCT	2 – 50 µM<br>100 mg/kg daily
Sonidegib (LDE225)	Active Biochem (Hongkong)	*In vitro*<br>*In vivo*	DMSO<br>MCT	2 – 50 µM<br>80 mg/kg daily
HhAntag	Genentech (San Francisco, USA)	*In vitro*<br>*In vivo*	DMSO<br>MCT	2 – 50 µM<br>100 mg/kg daily
Pictilisib (GDC-0941)	Genentech (San Francisco, USA)	*In vitro*<br>*In vivo*	DMSO<br>MCT	0,5 – 10 µM<br>75 mg/kg daily
PI-103	Alexis Biochemicals (San Diego, USA)	*In vitro*	DMSO	3 µM
MK-2206	Selleckchem (Munich, Germany)	*In vitro*	DMSO	5 µM
Everolimus	Sigma-Aldrich (St. Louis, USA)	*In vitro*	EtOH	50 nM
Rapamycin	Merck Millipore (Billerica, USA)	*In vitro*	DMSO	100 nM

SAG	Cayman Chemicals (Ann Arbor, USA)	*In vitro*	DMSO	100 nM

Supplementary Table S2: Oligonucleotides used for PCR.

**genotyping PCR**			
Primer Name	***Primer Sequence (5'-3' orientation)***	***mouse line***	***Amplification product***
Neo-R	GCATCAGAGCAGCCGATTGTCTG	*Ptch*$^{+/-}$	Neo-R & Exon 7-F: 950 bp (mutant allele)
Exon 7-F	AGGAAGTATATGCATTGGCAGGAG	*Ptch*$^{+/-}$	
mPTCwt_r	TCAAGGAGCAGAGGCCCAA	*Ptch*$^{+/-}$	mPTCwt_r & mPTCNx_f: 445 bp (wild type allele)
mPTCNx_f	GGGAGGGGATTTCAGCAGAATGTT	*Ptch*$^{+/-}$	

**qRT-PCR**				
Primer Name	***Primer Sequence (5'-3' orientation)***	***location***	***amplicon size***	***application***
mGli1-tq-f	TACATGCTGGTGGTGCACATG	exon 9	115 bp	*Gli1* expression
mGli1-tq-r	ACCGAAGGTGCGTCTTGAGG	exon 10		
HsaGli 1 tq F	AGCTACATCAACTCCGGCCA	exon 11	130 bp	*GLI1* expression
HsaGli1 tq R	GCTGCGGCGTTCAAGAGA	exon 12		
mHhipF.1	GGAGCCTTACTTGGACATTCACAA	*exon 4*	*143 bp*	*Hhip expression*

mHhipR.2	ACCGTTCCTGGTTGGTGGTATAA	*exon 5*		
HSA_Hhip_tq_FW1	ATGGTGGGTTGTGCTTTCCA	*exon 3*	*130 bp*	*HHIP expression*
HSA_Hhip_tq_RV1	CAGAAGCAGTTGTGTTTGTGCT	*exon 4*		
hsPTC1F.2	GAGGTTGGTCATGGTTACATGGA	*exon 6*	*196 bp*	*PTCH1 expression*
hsPTC1R.2	TGCTGTTCTTGACTGTGCCACC	*exon 7*		
18S-rev2	TTCCAATTACAGGGCCTCGAA	exon 1	81 bp	*18S rRNA* expression
18S-fwd	CGCAAATTACCCACTCCCG	exon 1		

Supplementary Table S3: Primary and secondary antibodies used for immunohistochemical and immunofluorescence analyses and western blot.

**primary antibody for IHC**	**dilution**	**antigen retrieval**
pAb mouse anti-Ki-67<br>BD, 556003	1:50	citric acid pH 6; 1x4 min & 4x3 min, microwave oven 600 W
**secondary antibody for IHC**	**dilution**	
En vision+ anti- rabbit/mouse/HRP*<br>Dako K5007	1:1	
**primary antibody for IF**	**dilution**	
mAB mouse anti-acetylated α-tubulin<br>Sigma Aldrich, T6793, clone 6-11B-1	1:500	
**secondary antibody for IF**	**dilution**	
pAB donkey anti-mouse-Alexa488<br>Dianova. 715-545-150	1:400	
**primary antibodies for Western Blot**	**dilution**	
mAb rabbit anti-Phospho-Akt (Ser473)<br>Cell Signaling, 4058	1:1000	
mAb mouse anti-Akt<br>BD, 610861	1:1000	
pAb rabbit anti-Caspase 3<br>Cell Signaling, 9962	1:500 (pro form)<br>1:250 (cleaved form)	
mAb rabbit anti-β-Actin<br>Cell Signaling, 4970	1:1000	

mAb mouse anti-HSC 70<br><br>Santa Cruz Biotechnology, sc-7298	1:10000
**secondary antibodies for Western Blot**	**dilution**
pAb goat anti-rabbit IgG HRP conjugated**<br><br>Dianova, 111-035-045	1:5000
pAb rabbit anti-mouse IgG HRP conjugated**<br><br>Dianova. 315-035-003	1:5000

* antibody binding was visualized using DAB+ (EnVision+ system-HRP, Dako).

** signals were visualized using the ECL plus detection system (GE Healthcare).

abbreviations: HRP, horseradish peroxidase; IHC, immunohistochemistry; IF: immunofluorescence; mAb, monoclonal antibody; pAb, polyclonal antibody.

1.2 Supplementary Figures

A

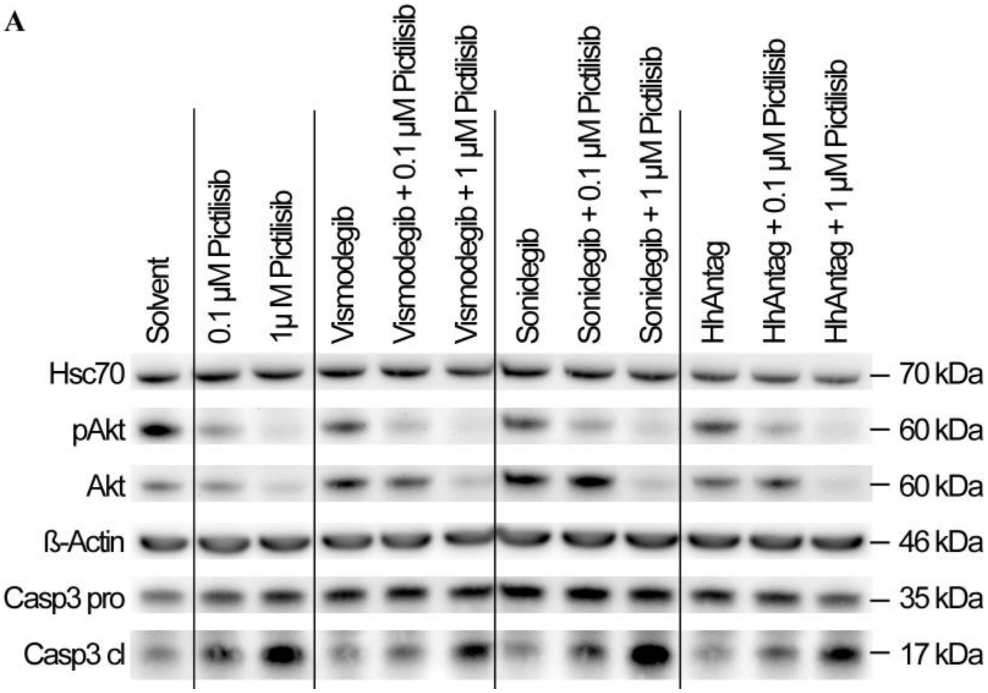

B

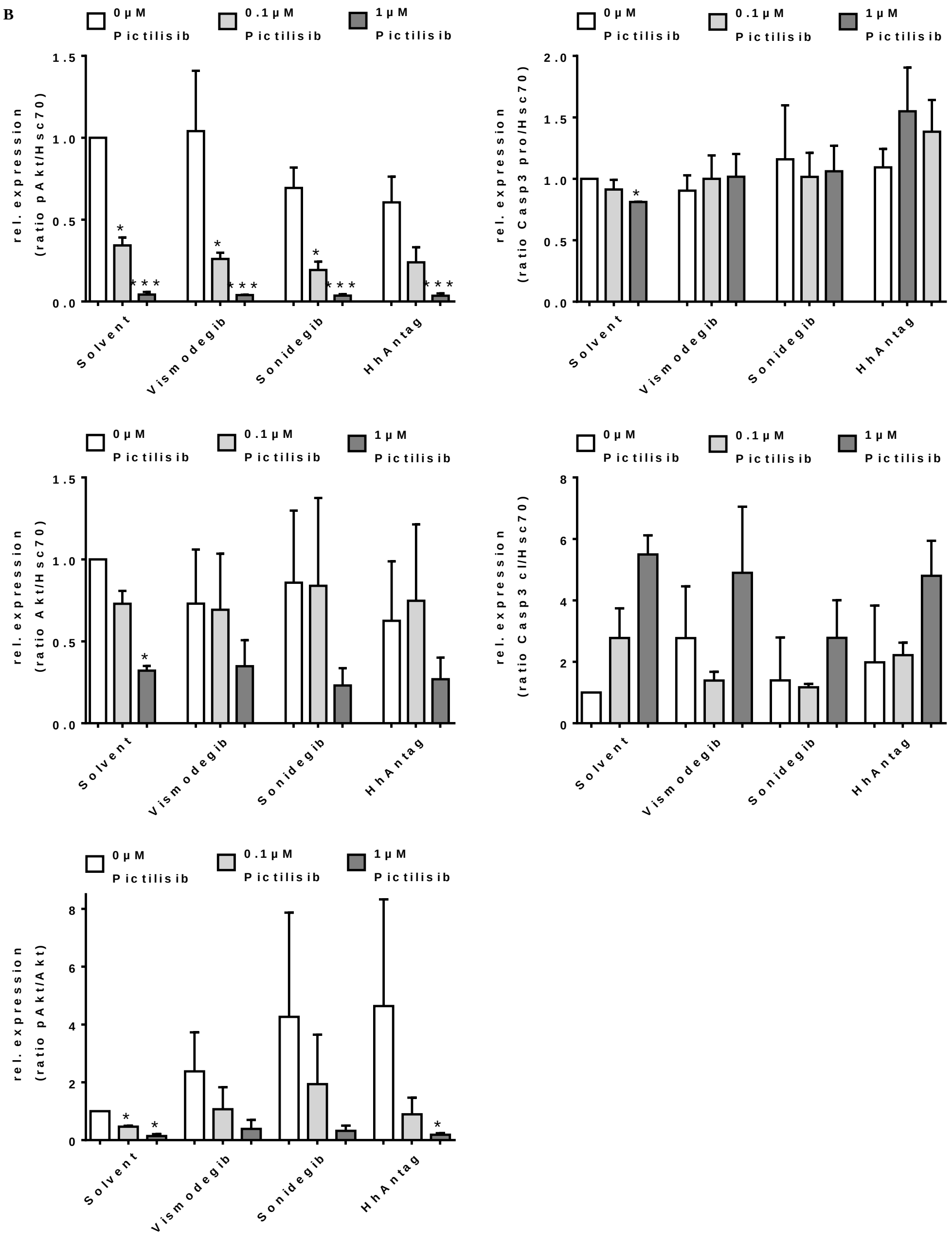

Supplementary Figure S1: Effects of Smo and/or Pi3k inhibition on Akt phosphorylation and caspase 3 cleavage of *Ptch* mutant ERMS cells. (A) Western blot analysis showing a representative blot out of three independent experiments. Cells were treated for 12 h with 5 µM vismodegib, 5 µM sonidegib, 5 µM HhAntag or 0.1 or 1 µM pictilisib, as indicated. **(B)** Relative pAkt and Akt protein expression levels and levels of caspase 3 proform and cleaved caspase 3 as measured by semiquantitative densitometry. For pAkt and Akt all three blots were quantified, for caspase 3 two out of three blots were quantified. Statistical analysis was done according to Tukey’s test for multiple comparisons. * $P < 0.05$ ** $P < 0.01$ *** $P < 0.001$ compared to solvent treated cells.

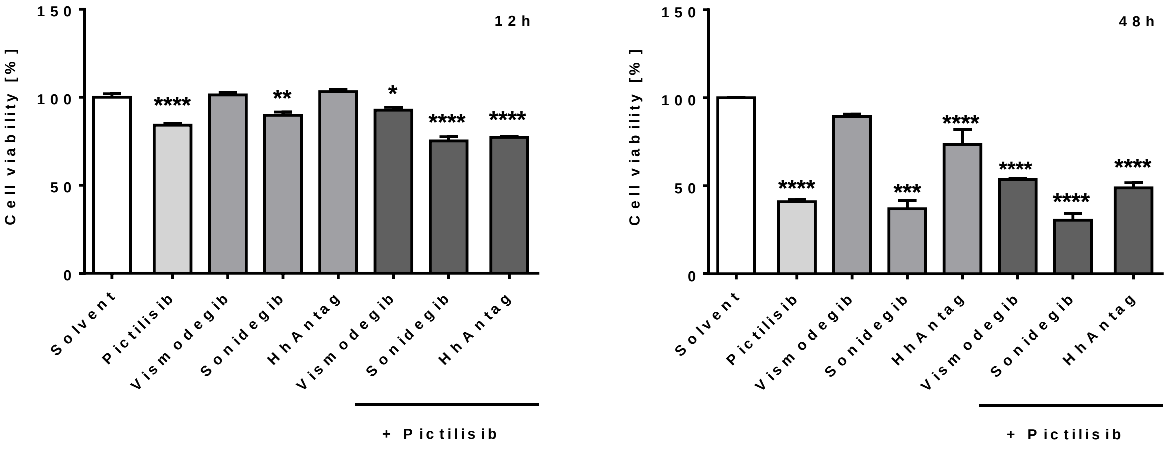

Supplementary Figure S2: Effects of SMO inhibitors and/or pictilisib on cell viability of *Ptch* mutant ERMS cells. Cell viability was measured by WST-1 assay. Cells were treated with SMO inhibitors for 12 h (left panel) or 48 h (right panel) and WST-1 was added for 4 h. The respective solvent controls were set to 100 %. Statistical analysis was done with One-Way-Anova. Bars represent the mean of six measurements + s.e. * P < 0.05, ** P < 0.01, *** P < 0.001, **** P < 0.0001 compared to solvent treated cells. Though the same drug concentrations were not toxic to human RMS cells (Ridzewski et al., 2015), they were toxic to primary ERMS cultures.

A

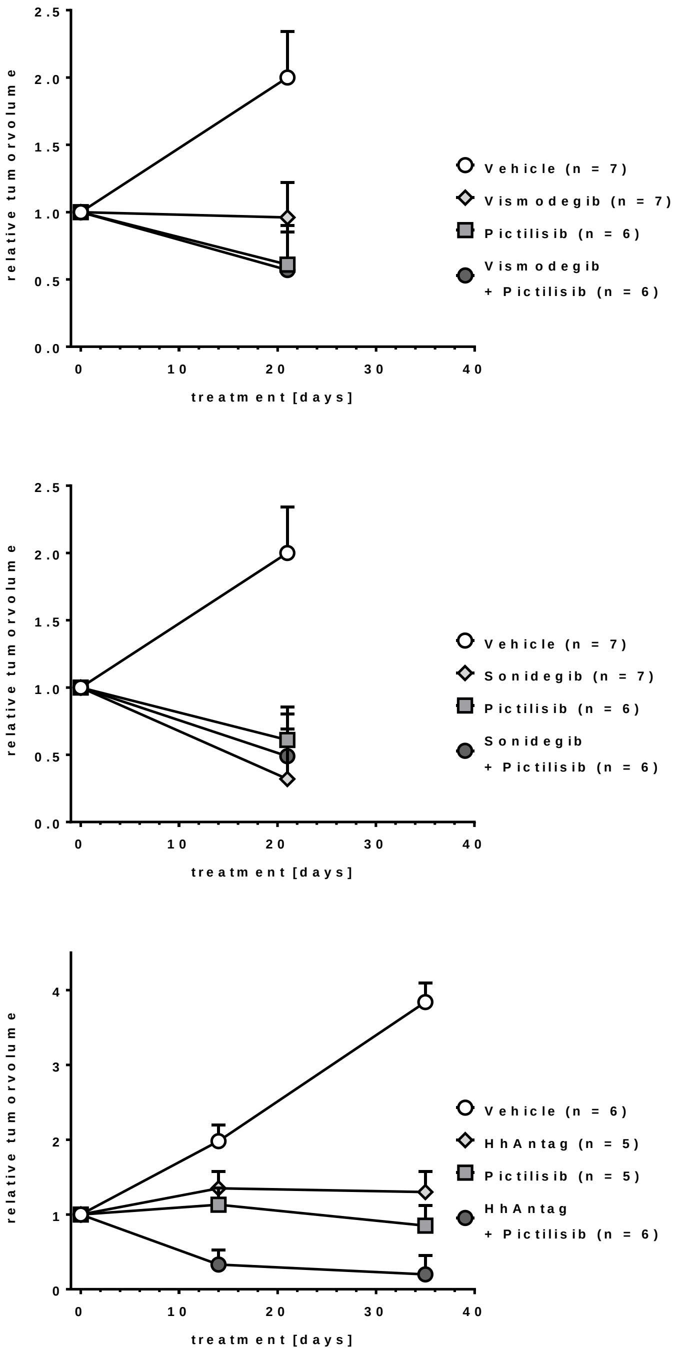

relative tumorvolume
treatment [days]
0.0
0.5
1.0
1.5
2.0
2.5
0
10
20
30
40
Vehicle (n = 7)
Vismodegib (n = 7)
Pictilisib (n = 6)
Vismodegib
+ Pictilisib (n = 6)
relative tumorvolume
treatment [days]
0.0
0.5
1.0
1.5
2.0
2.5
0
10
20
30
40
Vehicle (n = 7)
Sonidegib (n = 7)
Pictilisib (n = 6)
Sonidegib
+ Pictilisib (n = 6)
relative tumorvolume
treatment [days]
0
1
2
3
4
0
10
20
30
40
Vehicle (n = 6)
HhAntag (n = 5)
Pictilisib (n = 5)
HhAntag
+ Pictilisib (n = 6)

B

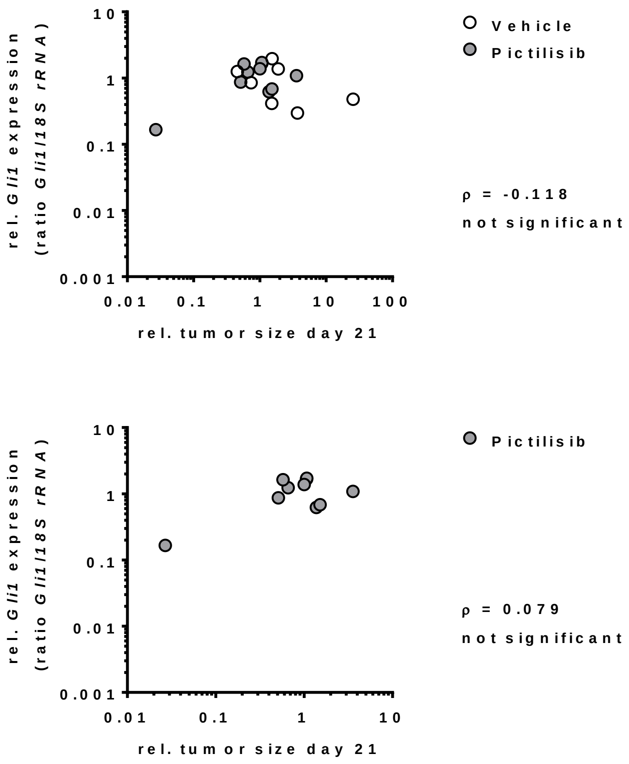

ρ : Spearman's rank correlation coefficient

C

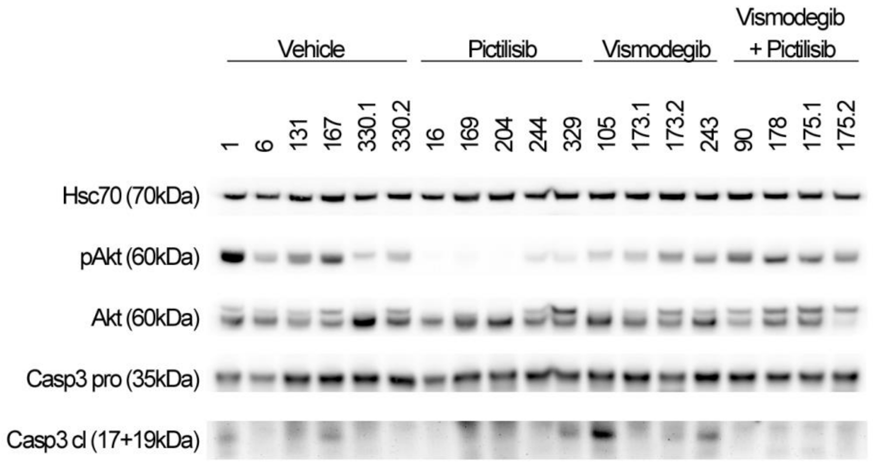
Vehicle
Pictilisib
Vismodegib
Vismodegib + Pictilisib
1 6 131 167 330.1 330.2 16 169 204 244 329 105 173.1 173.2 243 90 178 175.1 175.2
Hsc70 (70kDa)
pAkt (60kDa)
Akt (60kDa)
Casp3 pro (35kDa)
Casp3 cl (17+19kDa)

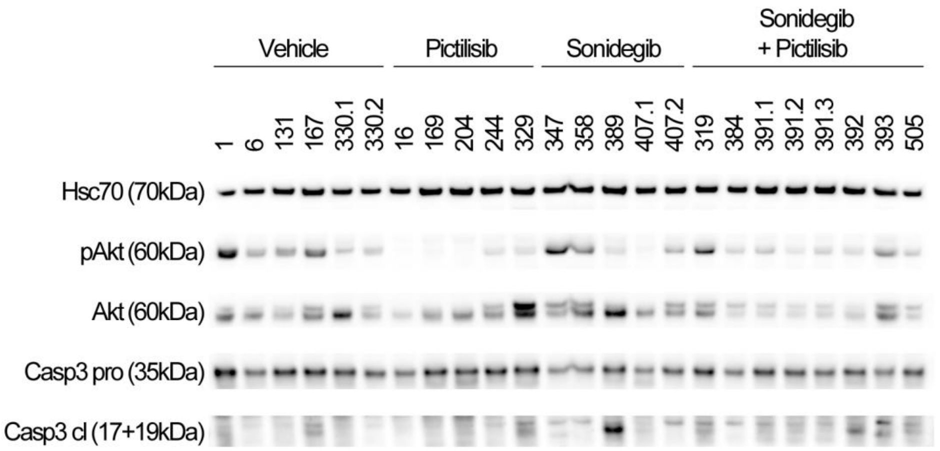
Vehicle
Pictilisib
Sonidegib
Sonidegib + Pictilisib
1 6 131 167 330.1 330.2 16 169 204 244 329 347 358 389 407.1 407.2 319 384 391.1 391.2 391.3 392 393 505
Hsc70 (70kDa)
pAkt (60kDa)
Akt (60kDa)
Casp3 pro (35kDa)
Casp3 cl (17+19kDa)

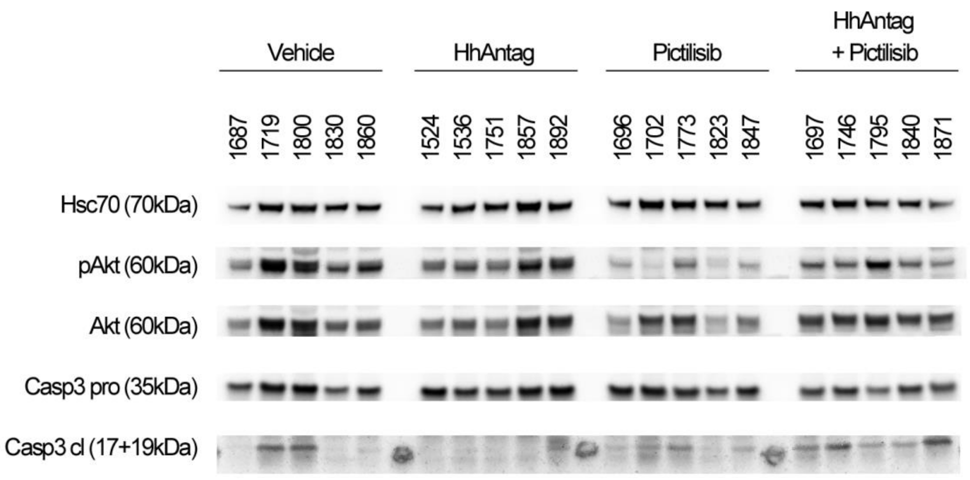
Vehicle
HhAntag
Pictilisib
HhAntag + Pictilisib
1687 1719 1800 1830 1860 1524 1536 1751 1857 1892 1696 1702 1773 1823 1847 1697 1746 1795 1840 1871
Hsc70 (70kDa)
pAkt (60kDa)
Akt (60kDa)
Casp3 pro (35kDa)
Casp3 cl (17+19kDa)

D

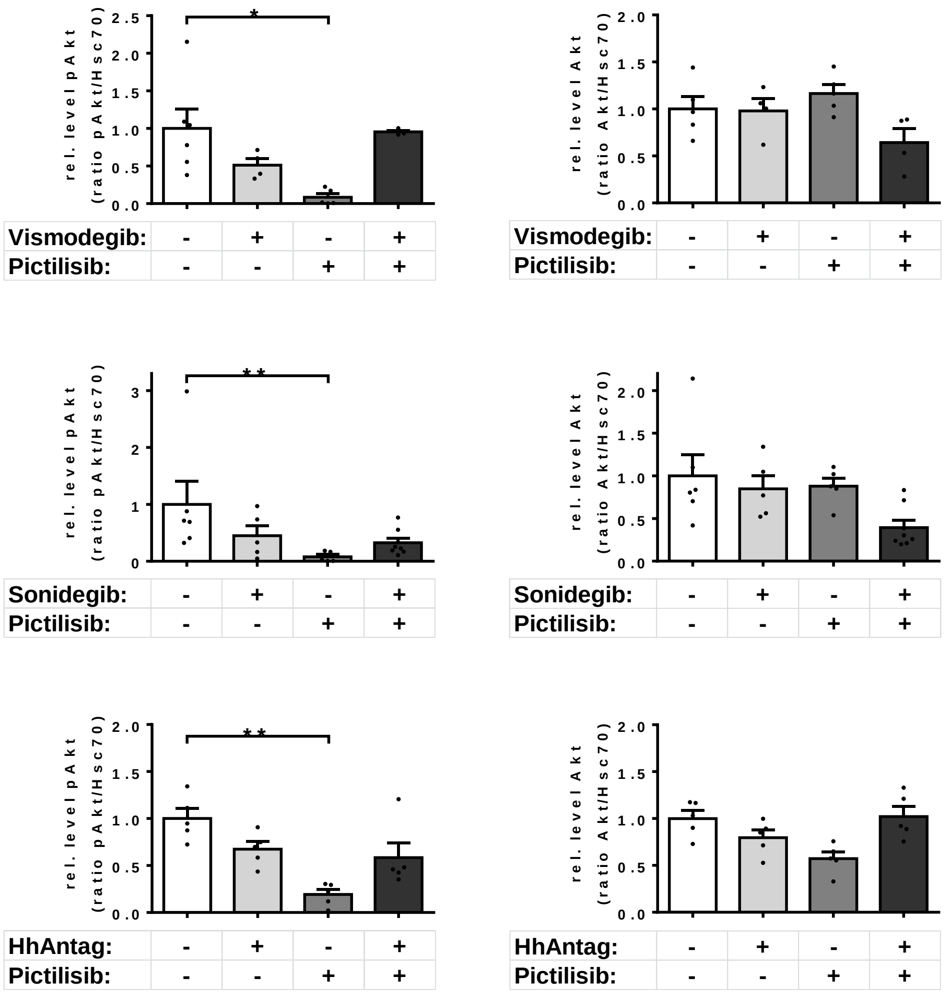

Supplementary Figure S3: Anticancer effects of SMO or PI3K inhibitors in ERMS-bearing *Ptch* mutant mice. (A) Tumor growth prediction model of the tumors shown in Fig. 2A. RMS-bearing mice received 100 mg/kg vismodegib, 80 mg/kg sonidegib or 100 mg/kg HhAntag alone or in combination with 75 mg/kg pictilisib daily for 21 (vismodegib/pictilisib or sonidegib/pictilisib) days or 35 (HhAntag/pictilisib) days by oral gavage. Relative logarithmic tumor volume was modeled in random effects mixed models with repeated volume measurements, adjusted for age at the start of treatment and sex. Multiple tumors of a mouse were considered as independent observations. The variance of tumor volume was allowed to vary between treatment regimes. **(B)** Spearman's rank correlation of *Gli1* expression with growth changes of tumors that have been treated with pictilisib or a combination of pictilisib and vehicle. **(C)** Representative Western blots (one out of at least two independent blots) showing the effect of vismodegib, sonidegib or HhAntag and/or pictilisib on Akt phosphorylation and caspase 3 cleavage in individual tumors (numbers indicate individual mice). Please note that cleavage of caspase 3 into the p17 and p12 active cleavage fragments was very difficult to detect. **(D)** Relative pAkt and Akt expression levels as measured by semiquantitative densitometry of Western blot data.

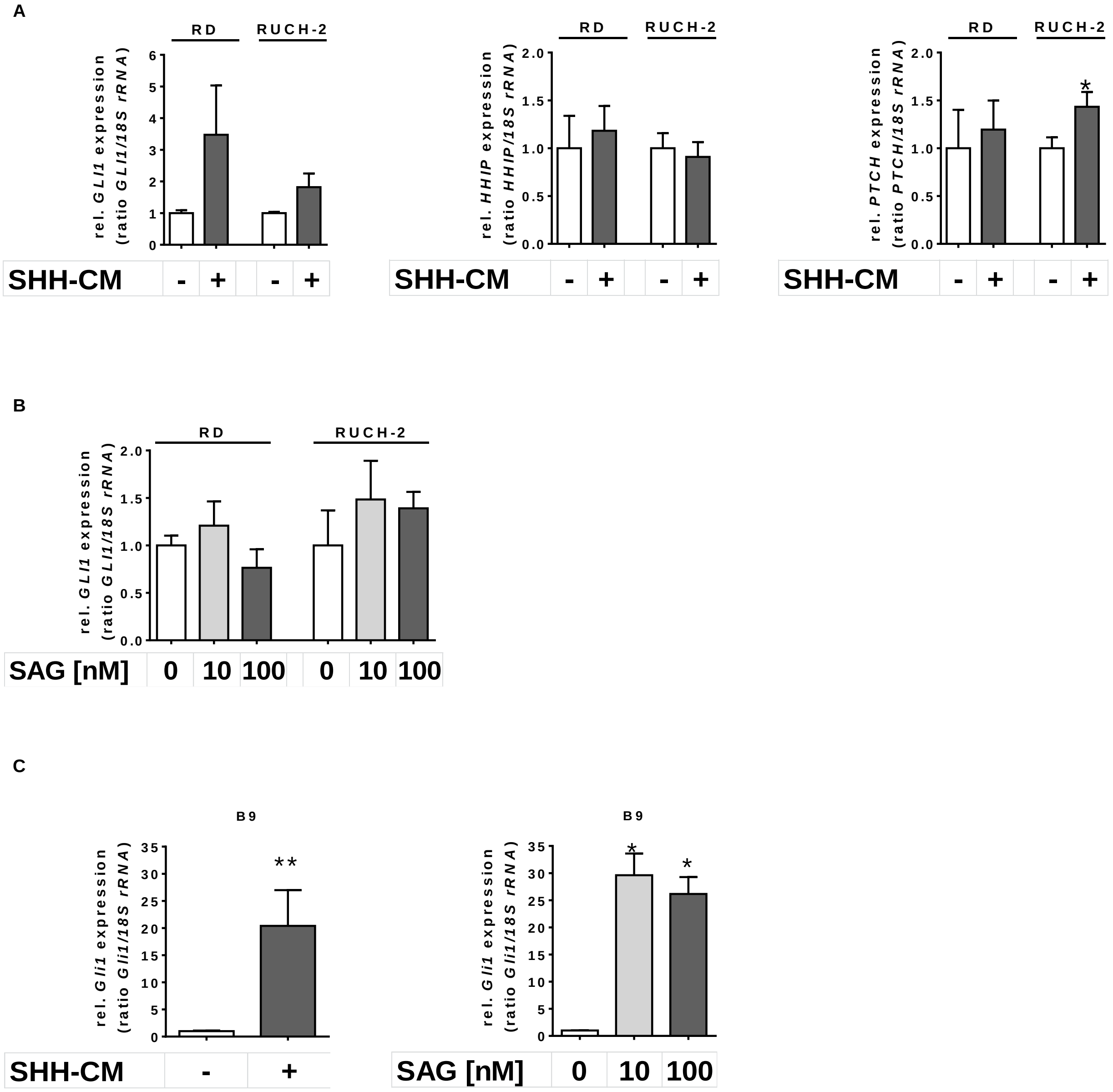
A
RD
RUCH-2
rel. GLI1 expression (ratio GLI1/18S rRNA)
rel. HHIP expression (ratio HHIP/18S rRNA)
rel. PTCH expression (ratio PTCH/18S rRNA)
SHH-CM
-
+
*
B
rel. GLI1 expression (ratio GLI1/18S rRNA)
SAG [nM]
0
10
100
C
B9
rel. Gli1 expression (ratio Gli1/18S rRNA)
**
*

D

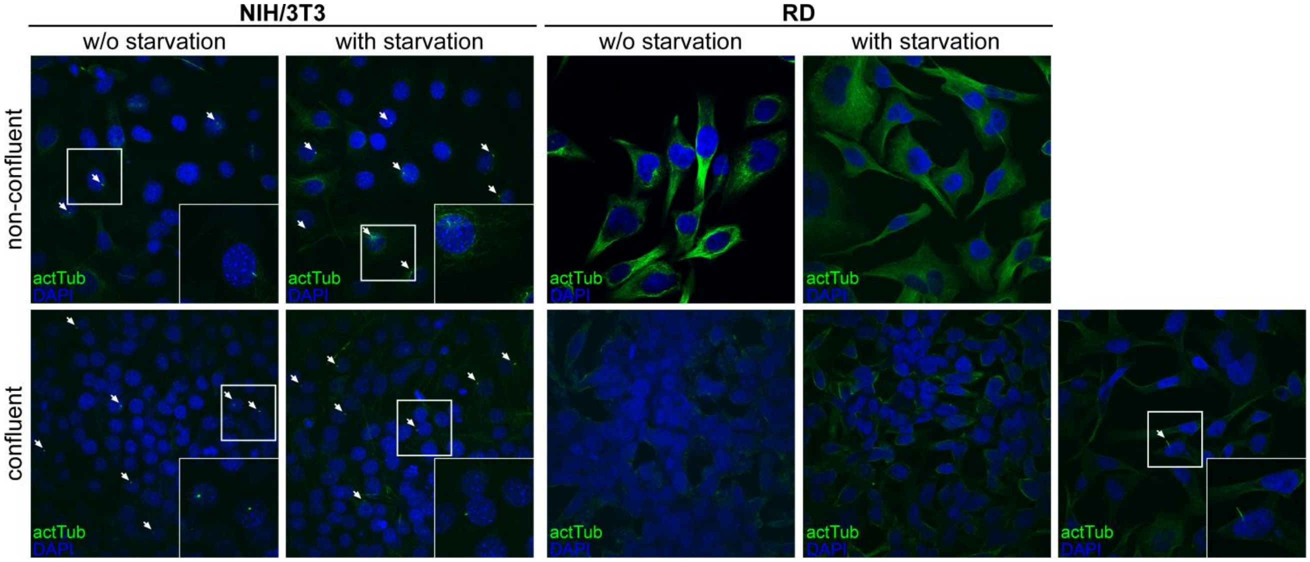

Supplementary Figure S4: Effects of SHH-conditioned medium and the SMO agonist SAG on HH signaling activity in human ERMS cell lines. (A) 24 h after seeding, RD and RUCH-2 cells were incubated with SHH conditioned medium (SHH-CM) or the respective control medium for 24 h. *GLI1*, *HHIP* and *PTCH* expression served as readout for HH signaling activity. **(B)** *GLI1* expression in RD and RUCH-2 cells upon incubation with SAG for 24 h. Bars represent the mean +s.e. of two independent experiments performed in duplicates. Statistical analysis was done according to Dunnett's test for multiple comparisons. **(C)** The functionality of SHH-CM and SAG was tested on Shh-responsive murine B9 fibroblasts that are highly responsive to SHH (Uhmann et al., 2011). Statistical analysis was done by One-Way-Anova for SAG treatments or by unpaired t-test for SHH-CM treatment. * $P < 0.05$ ** $P < 0.01$ compared to vehicle-treated or control-CM-treated cells, respectively. **(D)** Under the same conditions (non-confluent, without starvation) RD and RUCH-2 have not developed cilia whereas NIH/3T3 did. Extremely rarely single cilia are found on RD cells at the confluent stage (and also on RUCH-2 cells; data for RUCH-2 cells not shown). Fluorescent stainings were documented on a confocal laser scanning microscope equipped with software Fluoview FV100 (Olympus Corporation). Pictures were captured at 600-fold magnification, the insets at 3000-fold magnification. White arrows point to cilia.

A

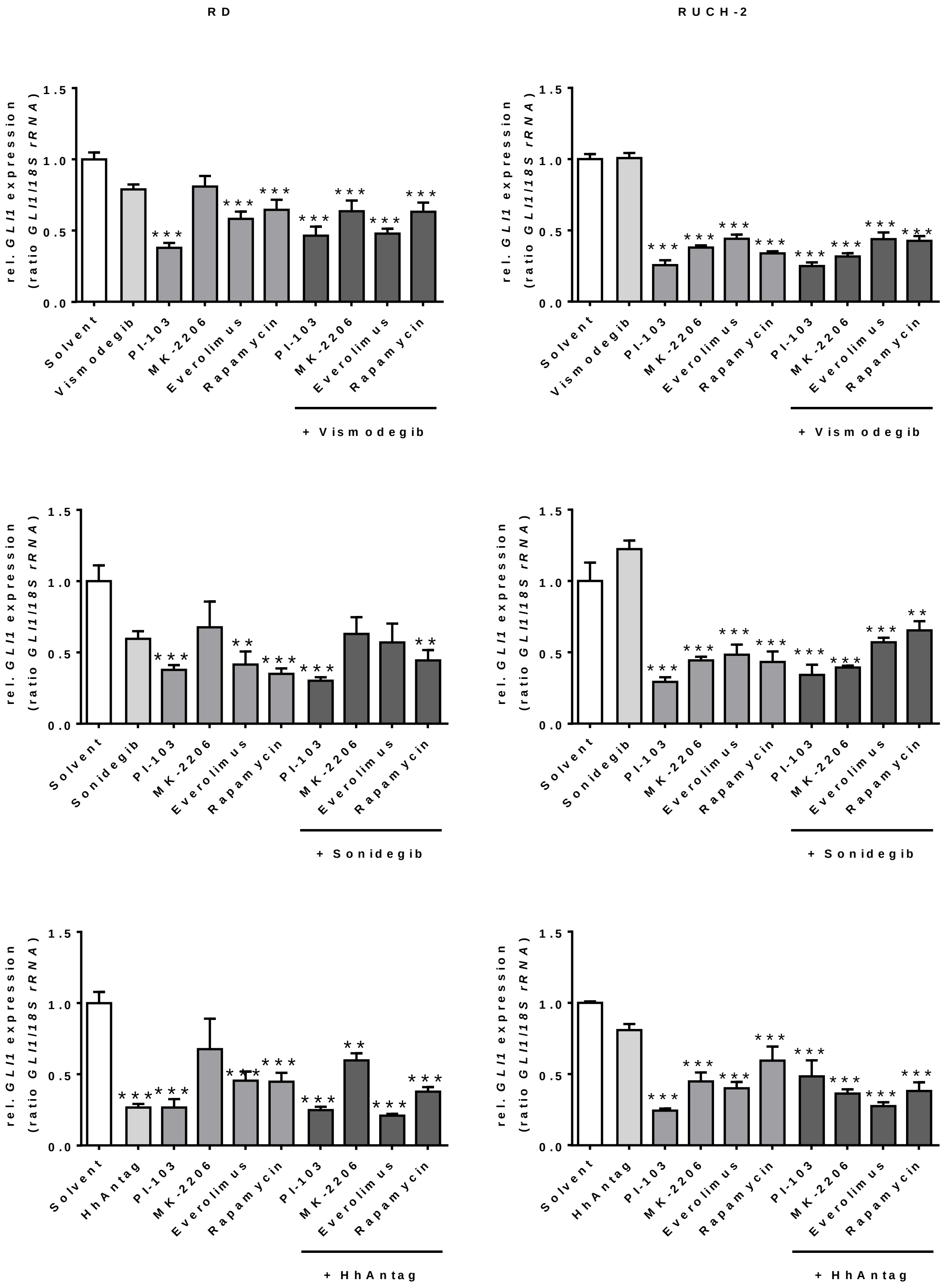
RD
RUCH-2
rel. *GLI1* expression
(ratio *GLI1*/*18S rRNA*)
1.5
1.0
0.5
0.0
Solvent
Vismodegib
PI-103
MK-2206
Everolimus
Rapamycin
+ Vismodegib
Sonidegib
+ Sonidegib
HhAntag
+ HhAntag
***
**

B

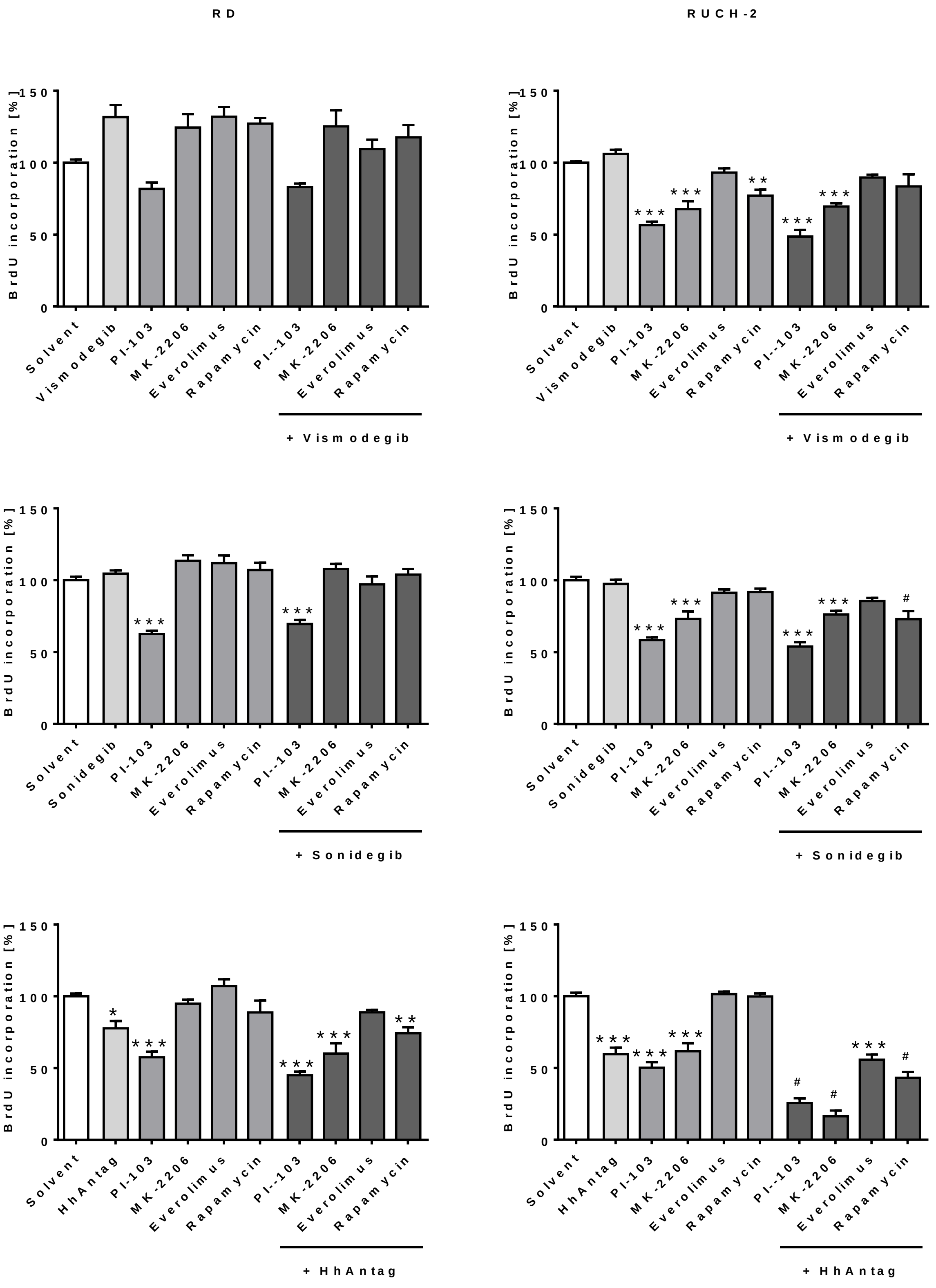
RD
RUCH-2
BrdU incorporation [%]
0
50
100
150
Solvent
Vismodegib
PI-103
MK-2206
Everolimus
Rapamycin
PI--103
+ Vismodegib
Sonidegib
+ Sonidegib
HhAntag
+ HhAntag
*
**
***
#

C

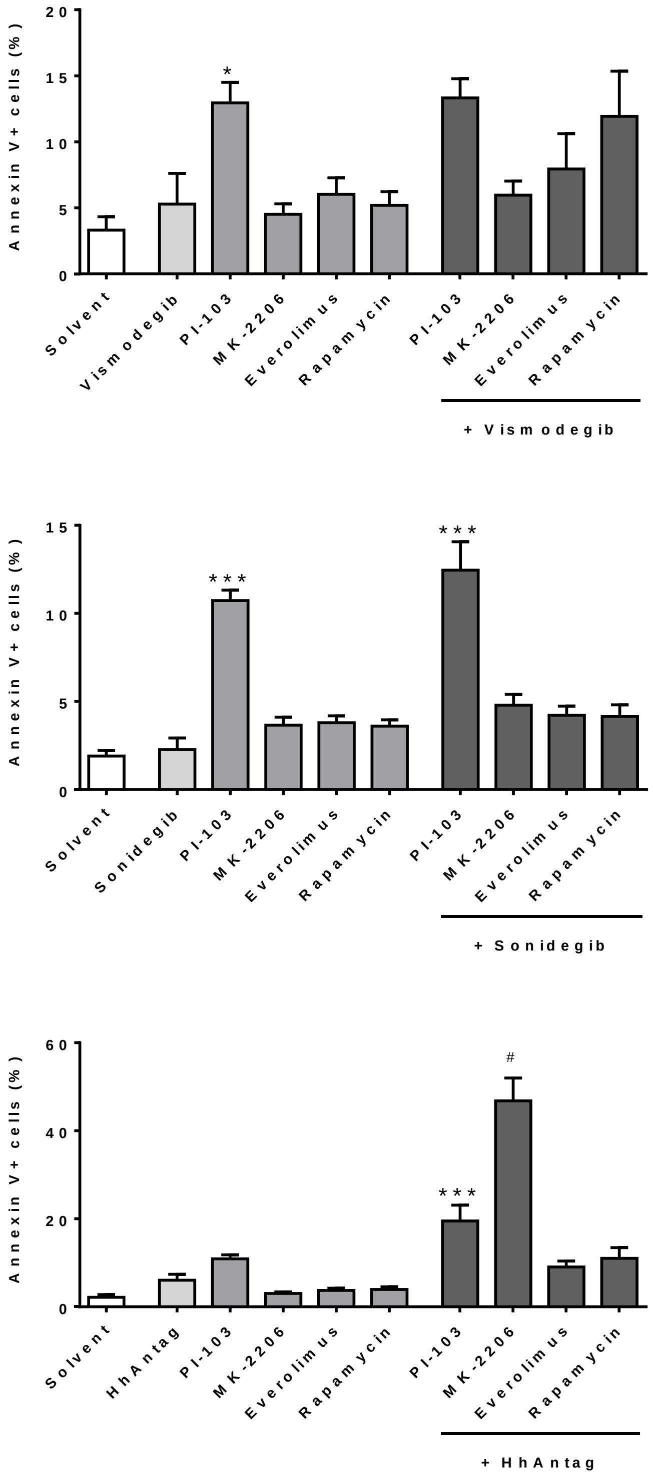
Annexin V+ cells (%)
0
5
10
15
20
*
Solvent
Vismodegib
PI-103
MK-2206
Everolimus
Rapamycin
PI-103
MK-2206
Everolimus
Rapamycin
+ Vismodegib
Annexin V+ cells (%)
0
5
10
15
***
***
Solvent
Sonidegib
PI-103
MK-2206
Everolimus
Rapamycin
PI-103
MK-2206
Everolimus
Rapamycin
+ Sonidegib
Annexin V+ cells (%)
0
20
40
60
***
#
Solvent
HhAntag
PI-103
MK-2206
Everolimus
Rapamycin
PI-103
MK-2206
Everolimus
Rapamycin
+ HhAntag

Supplementary Figure S5: Effects of SMO and/or PI3K/AKT/mTOR inhibitors on HH signaling activity, proliferation and apoptosis in cell lines derived from sporadic ERMS. RD and RUCH-2 cells treated with 10 µM of SMO inhibitors and/or the dual PI3K/mTOR inhibitor PI-103, the pure AKT inhibitor MK-2206 or the mTOR inhibitors everolimus and rapamycin. Concentrations of PI3K/AKT/mTOR inhibitors were adapted from the appropriate literature and were 3 µM, 5 µM, 50 nM and 100 nM, respectively. **(A)** *GLI1* expression levels after treatment for 24 h. **(B)** BrdU incorporation after treatment for 24 h. BrdU incorporation of solvent treated cells was set to 100 %. Bars represent the mean +s.e. of three independent experiments performed in triplicates. **(C)** Annexin V staining and subsequent FACS analysis of RD cells treated for 48 h with the drugs as indicated. Bars represent the mean number of Annexin V^+ cells +s.e. of two independent experiments performed in duplicates.* $P < 0.05$, ** $P < 0.01$, *** $P < 0.01$ compared to cells treated with solvent and analyzed by Tukey's test for multiple comparisons.

RD **RUCH-2**

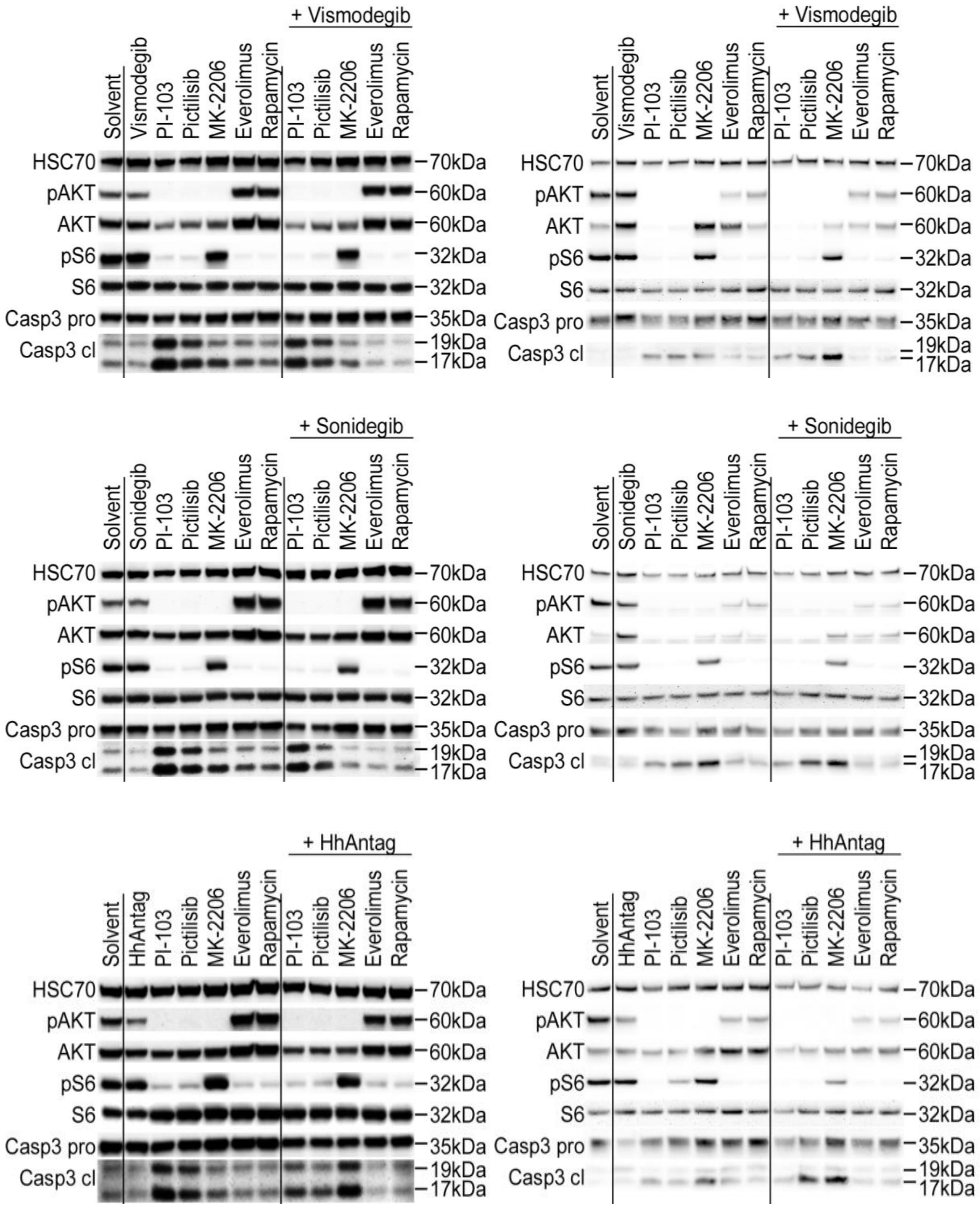

+ Vismodegib
Solvent
Vismodegib
PI-103
Pictilisib
MK-2206
Everolimus
Rapamycin
HSC70
pAKT
AKT
pS6
S6
Casp3 pro
Casp3 cl
70kDa
60kDa
60kDa
32kDa
32kDa
35kDa
19kDa
17kDa
+ Sonidegib
Sonidegib
+ HhAntag
HhAntag

Supplementary Figure S6: Effects of SMO and/or PI3K/AKT/mTOR inhibition on phosphorylation of AKT, S6 and caspase cleavage in human ERMS cell lines. Western blot of RD and RUCH-2 cells treated for 48 h with vismodegib, sonidegib or HhAntag alone or in combination with PI-103, pictilisib, MK-2206, everolimus or rapamycin. Vismodegib or sonidegib did not evoke any obvious effects on pAKT or pS6/S6 levels in RD and RUCH-2 cells, but seem to enhance the total level of AKT in RUCH-2 cells. As already described by our lab (Ridzewski et al., 2015), HhAntag reduced pAKT in RD and RUCH-2 cells, but did not affect AKT or pS6/S6 levels. None of the SMO inhibitors affected caspase 3 cleavage. PI-103, pictilisib and MK-2206 efficiently reduced pAKT in RD and RUCH-2 cells. PI-103 and pictilisib, but not MK-2206, additionally reduced pS6. Everolimus and rapamycin reduced pS6, as expected, in both cell lines but enhanced pAKT in RD. Furthermore PI-103, pictilisib and MK-2206 induced cleavage of caspase 3 in RD and RUCH-2 cells.

In RD cells, the combination of vismodegib, sonidegib and HhAntag with PI3K/AKT/mTOR inhibitors did not change the protein pattern. The only and obvious exception was the combination of HhAntag plus MK-2206, which induced cleavage of caspase 3.

Similarly, in RUCH-2 cells many of the combinations did not enhance the effects that were observed upon treatment with either drug alone. However, exceptions were the combination of vismodegib plus MK-2206 that reduced the protein level of AKT and the combinations of HhAntag plus pictilisib and HhAntag plus MK-2206 that reduced the level of pS6 more efficiently than either single drug alone. In addition the combinations of vismodegib plus MK-2206 and HhAntag plus pictilisib more efficiently induced cleavage of caspase 3 when compared to the single drugs.

References

Ridzewski, R., Rettberg, D., Dittmann, K., Cuvelier, N., Fulda, S., and Hahn, H. (2015). Hedgehog Inhibitors in Rhabdomyosarcoma: A Comparison of Four Compounds and Responsiveness of Four Cell Lines. *Front Oncol* 5**,** 130. doi: 10.3389/fonc.2015.00130.

Uhmann, A., Niemann, H., Lammering, B., Henkel, C., Hess, I., Nitzki, F., Fritsch, A., Prufer, N., Rosenberger, A., Dullin, C., Schraepler, A., Reifenberger, J., Schweyer, S., Pietsch, T., Strutz, F., Schulz-Schaeffer, W., and Hahn, H. (2011). Antitumoral effects of calcitriol in basal cell carcinomas involve inhibition of hedgehog signaling and induction of vitamin D receptor signaling and differentiation. *Mol Cancer Ther* 10**,** 2179-2188. doi: 10.1158/1535-7163.MCT-11-0422.
